# A versatile nanoplatform for enhancing the therapeutic efficacy against low-immunogenic TNBC by inducing immunogenic cell death and MHC-I upregulation

**DOI:** 10.1016/j.mtbio.2026.102927

**Published:** 2026-02-10

**Authors:** Shanlingzi Huang, Lu Gao, Yujun Chen, Zhaoming Fu, Ziyou Wang, Yifan Liu, Zhicheng Zhou, Ru Huang, Wen Song, Feifan Zhou

**Affiliations:** a State Key Laboratory of Digital Medical Engineering, School of Biomedical Engineering, Hainan University, Sanya, 572025, China; b Key Laboratory of Biomedical Engineering of Hainan Province, One Health Institute, Hainan University, Sanya, 572025, China

**Keywords:** Photodynamic therapy, Mitochondrial electron transport chain, Low immunogenicity, Antigen presentation, MHC-I upregulation

## Abstract

Triple-negative breast cancer (TNBC), characterized by low immunogenicity, is a challenging issue in clinical treatment due to its poor response to various therapies. The transformation of TNBC from a “cold tumor” with low immunogenicity into a “hot tumor” that elicits stronger immune responses is a key research focus. Photodynamic therapy (PDT) has emerged as a promising solution for TNBC treatment because it can effectively induce immunogenic cell death (ICD), which prompts the release of damage-associated molecular patterns (DAMPs) and activates immune responses. The role of MHC-I molecules in antigen presentation is crucial, but TNBC cells often evade immune surveillance by downregulating or losing MHC-I expression. Recent studies have shown that inhibiting the activity of complex II (CII) in the mitochondrial electron transport chain of tumor cells can promote MHC-I expression. Based on this finding, the combination of the PDT photosensitizer PCN-224 with the CII inhibitor 3-nitropropionic acid (3NPA) to form PCN@3NPA has been innovatively developed. Furthermore, modification with hyaluronic acid (HA) enables targeted delivery to TNBC cells that overexpress CD44. PDT induces ICD in tumor cells, releasing large amounts of DAMPs, while the sustained release of 3NPA effectively inhibits CII activity, significantly enhances MHC-I expression, thereby boosting tumor cell immunogenicity. This process significantly improves the recognition and killing of tumor cells by CD8^+^ T cells, markedly strengthening the body's anti-tumor immune response. This approach offers a novel and promising strategy for efficiently treating not only low-immunogenic TNBC but also other similar tumors, bringing new hope for future clinical treatments.

## Graphical abstract

The study presents an innovative strategy to enhance the immunogenicity of TNBC, a challenging cancer subtype with low immunogenicity and poor prognosis. By inducing immunogenic cell death (ICD) and upregulating MHC-I expression, the approach aims to transform TNBC from a “cold tumor” to a “hot tumor,” thereby improving its responsiveness to immunotherapy.

**Figure:**
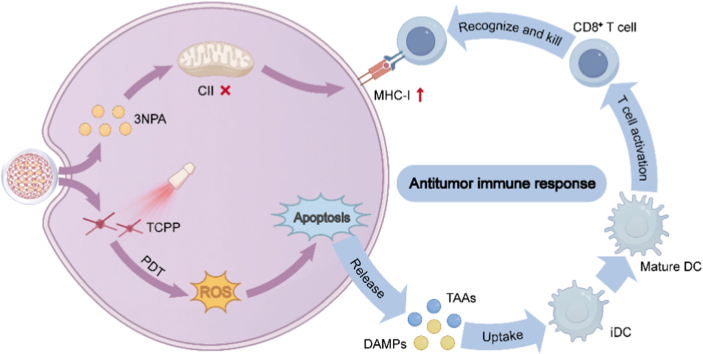

## 1 Introduction

In recent years, the development of cancer immunotherapy has brought a ray of hope to cancer patients. For example, immune checkpoint blockade (ICB)-based tumor immunotherapy has demonstrated excellent and durable clinical efficacy in various malignant tumors [[1], [2], [3]], including melanoma [4]. This efficacy is achieved by blocking the immune escape mechanisms of tumor cells and activating the activity of tumor-specific T cells. However, certain tumor subtypes, such as triple-negative breast cancer (TNBC) [5,6], exhibit low immunogenicity and are considered “cold” tumors [7,8]. This characteristic is one of the main reasons why the majority of cancer patients have a low response to multiple therapeutic approaches, including immunotherapy. Therefore, the development of a strategy to transform “cold” tumors into “hot” tumors, that is, to enhance the immunogenicity of cancer cells, holds significant importance for the clinical development of anti-tumor therapies.

Photodynamic therapy (PDT) is a novel cancer treatment that has gained a lot of traction in recent years because of its minimal invasiveness, low toxicity, and high spatiotemporal selectivity [9,10]. Extensive studies [[11], [12], [13]] have shown that PDT can not only directly destroy tumor cells but also activate the body's immune response by inducing immunogenic cell death (ICD). The induction of ICD [14,15] is a unique form of cell death, characterized by the release of damage-associated molecular patterns (DAMPs) from dying tumor cells, including calreticulin (CRT) and high-mobility group box 1 (HMGB1). The release of these molecules facilitates the maturation and activation of dendritic cells (DCs) [[16], [17], [18]], thereby triggering a systemic anti-tumor immune response. In this way, tumor cells are directly killed by PDT, and an in situ autologous immunogenic vaccine is generated [19,20], releasing tumor antigens to activate the immune response [[21], [22], [23]].

It is worth noting that in the complex process of immune responses, antigen presentation is a key step in immune activation [24,25]. During this process, the major histocompatibility complex class I (MHC-I) molecules play a crucial role [[26], [27], [28]]. The collaboration between MHC-I molecules and DCs can be regarded as the critical “spark” that ignites the anti-tumor immune response. Specifically, the presentation of tumor antigens by MHC-I molecules on DCs is essential for the activation of CD8^+^ T cells [29,30]. This process is analogous to providing a “key” that activates their subsequent proliferation and differentiation, resulting in the generation of a large number of cytotoxic T lymphocytes (CTLs) [31,32]. These CTLs are capable of specifically recognizing and eliminating tumor cells that express the corresponding antigens. The activation of CD8^+^ T cells lead to their migration to the tumor site, where they use their specific receptors to precisely target and eliminate these tumor cells. It has been shown that to evade immune surveillance and attack, TNBC, which is an immunogenicity-deficient “cold tumor”, often employs various mechanisms [33,34] to downregulate or completely lose MHC-I expression. This allows TNBC to escape detection by the immune system. Therefore, the development of a strategy to upregulate MHC-I expression holds significant guiding importance for the treatment of TNBC. Additionally, exploring its synergistic effects with PDT-mediated ICD is crucial [[35], [36], [37]].

**Scheme 1:**
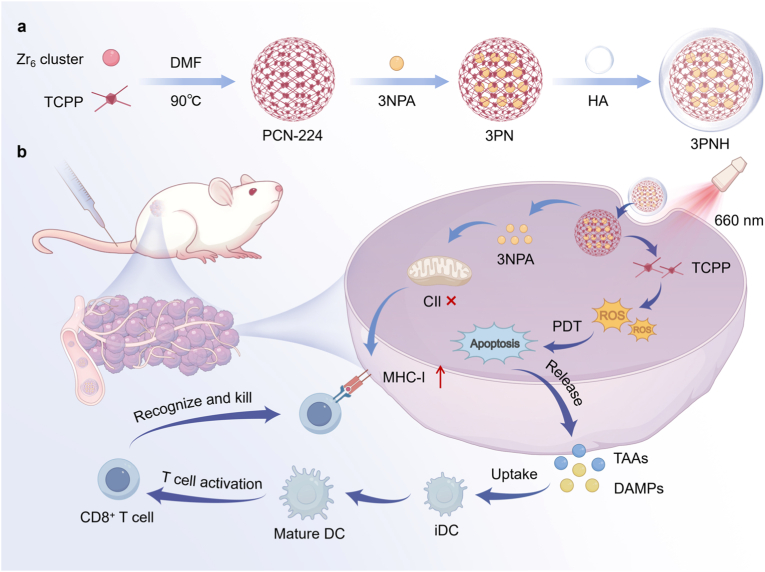
(a) Schematic illustration of the synthesis process of 3PNH. (b) After intravenous injection, 3PNH accumulates in the tumor region through HA-mediated targeting. On one hand, upon irradiation with 660 nm laser, 3PNH generates ROS, which induce ICD in tumor cells, leading to the release of tumor-associated antigens. This process promotes the maturation of DCs and activates T lymphocytes. On the other hand, the release of 3NPA from 3PNH within tumor cells inhibits the activity of mitochondrial CII, thereby enhancing the expression of MHC-I on tumor cells. This further strengthens the recognition and killing of tumor cells by CD8^+^ T cells.

Recently, it has been found that inhibiting the activity of Complex II (CII) in the mitochondrial electron transport chain in tumor cells can cause an accumulation of the metabolic byproduct succinate [38]. This change promotes the expression of major histocompatibility complex antigen processing and presentation genes (MHC-APP), which ultimately leads to increased expression of MHC-I on the surface of tumor cells. Based on these findings, a combined therapeutic strategy targeting low-immunogenic TNBC has been developed to enhance MHC-I expression in tumor cells and synergize with PDT. As shown in Scheme 1a, PCN-224 was selected as the photosensitizer for PDT, and the CII inhibitor, 3-nitropropionic acid (3NPA), was loaded into the cavities of PCN-224 to form PCN@3NPA (3 PN). Hyaluronic acid (HA) was then used to modify the outermost layer of 3 PN, endowing PCN@3NPA@HA (3PNH) with CD44 targeting capabilities, as TNBC cells overexpress CD44. Through HA-mediated tumor targeting, 3PNH precisely and actively reaches the tumor region. Subsequently, PDT mediated by PCN-224 initiates the first round of tumor cell killing. Concurrently, the release of 3NPA inhibits the activity of CII in tumor cells, leading to increased expression of MHC-APP and ultimately enhancing MHC-I protein expression. At this point, the immunogenicity of tumor cells is significantly elevated, and the recognition and killing ability of CD8^+^ T cells against tumor cells are markedly enhanced. This significantly strengthens the antitumor immune response and improves antitumor efficacy (Scheme 1b). The development of this strategy provides a new approach for the efficient treatment of low-immunogenic tumors.

## 2 Materials and methods

### 2.1 Materials

Meso-tetra(4-carboxyphenyl)porphine (TCPP) was provided by Shanghai Macklin Biochemical Co., Ltd. (Shanghai). Zirconium oxychloride octahydrate (ZrOCl_2_·8H_2_O), benzoic acid (BA), N, N′-dimethylformamide (DMF) and 3-nitropropionic acid (3NPA) were purchased from Aladdin Company, Sinopharm Chemical Reagent Co., Ltd., and Xilong Scientific Co., Ltd., respectively. 2′,7′-dichlorodihydrofluorescein diacetate (DCFH-DA) and 4T1 cells were obtained from Yisheng Biotech and Shanghai Ausail Biotechnology Co., Ltd., respectively. Calcein-AM/PI (cell viability and cytotoxicity detection kit), CCK-8 kit, Hoechst 33342, mitochondrial membrane potential detection kit (TMRE), and Annexin V-FITC apoptosis detection kit were all purchased from Beyotime Biotechnology Co., Ltd. The RNA extraction kit and the qPCR-specific reverse transcription kit were obtained from Sangon Biotech (Shanghai) Co., Ltd. and Bio-Rad Laboratories (Beijing) Co., Ltd., respectively. Cell staining buffer, anti-CD11c-FITC, anti-CD86-APC, anti-CD80-PE, anti-CD3-FITC, anti-CD8-APC, anti-CD69-PE, anti-CD4-AF700, anti-MHC-I-FITC, anti-CD107a-PE and anti-CD69-APC antibodies, and ELISA kits were all purchased from Biolegend Inc. (USA).

### 2.2 Release kinetics of 3NPA

1 mL of 3PNH was placed into a dialysis bag with a molecular weight of 3.5 kDa. The dialysis bag was immersed in 50 mL of HEPES buffer (pH 7.4), with one setup containing ATP (5 mg/mL) and the other without ATP. The drug release experiment was conducted in a shaking incubator at 37 °C with a speed of 200 rpm. At predetermined time points (0, 0.5, 1, 2, 4, 6 h), 1.5 mL of the dialysate was withdrawn and immediately replaced with the same volume of fresh buffer. The cumulative release of 3NPA in the dialysate was quantitatively analyzed using high-performance liquid chromatography-mass spectrometry (HPLC-MS).

### 2.3 Detection of ROS in solution

The reactive oxygen species (ROS) generation capacity of the nanomaterials was evaluated using the DCFH-DA probe. The evaluation was conducted as follows: Initially, 1 μL of a 10 mM DCFH-DA solution was added to 499 μL of PBS solution that had been treated with 0.01 M NaOH for 0.5 h, resulting in the conversion of DCFH-DA to DCFH with a final concentration of 20 μM. Subsequently, 25 μL of the 20 μM DCFH-DA probe was mixed with PBS (975 μL), 3NPA (0.25 mM, 975 μL), PH (10 μg/mL, 975 μL), and 3PNH (10 μg/mL, 975 μL), respectively. Under irradiation with a 660 nm laser (0.5 W/cm^2^), the fluorescence intensity of the solutions at 525 nm (excitation wavelength: 488 nm) was measured every 30 s. The changes in fluorescence intensity allowed for real-time monitoring of the ROS generation capacity of the nanomaterials under light exposure, thereby assessing their activity in photocatalytic reactions.

### 2.4 *in vitro* reactive oxygen species (ROS) detection

***Flow Cytometry Analysis:*** 4T1 cells were seeded in 12-well plates at a density of 2 × 10^5^ cells per well and cultured for 24 h. Co-culture with PH (2 μg/mL), 3PNH (2 μg/mL), and 3NPA (0.25 mM) for 6 h was then performed, respectively. After three washes with PBS, staining with DCFH-DA (5 μM) for 30 min was carried out, followed by irradiation with a 660 nm laser (0.5 W/cm^2^) for 5 min. Intracellular ROS levels were detected using flow cytometry (Beckman CytoFLEX).

***Confocal Microscopy Observation:*** 4T1 cells were seeded in confocal culture dishes at a density of 3 × 10^5^ cells per well and treated with the same methods as described above. Staining with Hoechst 33342 (10 μg/mL) for 15 min was performed to label the nuclei. Fluorescence signals were observed using confocal laser scanning microscope (CLSM, Olympus FV3000). The excitation/emission wavelengths for DCFH were 488/525 nm, and those for Hoechst were 350/461 nm.

### 2.5 Mitochondrial membrane potential detection

Changes in mitochondrial membrane potential were detected using the tetramethylrhodamine ethyl ester (TMRE) fluorescent probe. Co-incubation of 4T1 cells with PBS, 3NPA (6 μg/mL), PH (3 μg/mL), and 3PNH (3 μg/mL) for 6 h was performed, respectively. After irradiation with a laser for 5 min, the cells were further cultured for 12 h. Following three washes with PBS, staining with TMRE for 20 min and Hoechst 33342 for 15 min was carried out. Fluorescence intensity changes were observed using CLSM. The excitation/emission wavelengths for TMRE were 550/575 nm.

### 2.6 Construction of 3D tumor spheroid model

A 96-well plate with a U-shaped bottom was pre-treated with anti-adhesive coating solution for 24 h 4T1 cells were seeded at a density of 1000 cells per well. After 48 h of static culture, 3D tumor spheroids were formed for subsequent efficacy evaluation assay.

### 2.7 Therapeutic efficacy evaluation of the 3D tumor spheroid

Culture media containing PBS, 3NPA (6 μg/mL), PH (3 μg/mL), or 3PNH (3 μg/mL) were added to the formed 3D tumor spheroids. The culture media were replaced every 48 h, and the volume changes of the 3D tumor spheroids were observed and measured using a microscope (MshOt). The volume of the tumor spheroids was calculated using the formula: *V* = length × width [2]/2​.

### 2.8 Flow Cytometry Analysis of MHC-I expression

4T1 cells were seeded at a density of 2 × 10^5^ cells per well in 12-well plates. After 24 h of culture, the cells were co-cultured with PBS, 3NPA (30 μg/mL), PH (15 μg/mL) or 3PNH (15 μg/mL) for 24 h. The cells were then washed three times with PBS, collected, and stained with H-2Kb/H-2Db antibodies. The expression levels of MHC-I molecules were detected by flow cytometry.

### 2.9 Evaluation of DCs maturation

Mouse bone marrow cells were collected and stimulated with RPMI-1640 medium containing GM-CSF (20 ng/mL) to induce differentiation into immature bone marrow-derived dendritic cells (BMDCs). These immature BMDCs were then co-cultured with the supernatant of 4T1 cells treated with various treatments for 24 h. After co-culture, the BMDCs were collected and stained with anti-CD11c-FITC, anti-CD86-APC, and anti-CD80-PE antibodies. The expression levels of surface markers CD11c, CD86, and CD80 on BMDCs were detected by flow cytometry to assess the maturation degree of BMDCs. Additionally, the levels of TNF-α, IL-6, and IFN-γ in the co-culture supernatant were measured by ELISA to further evaluate the activation of BMDCs.

### 2.10 Evaluation of T cell activation

BMDCs were collected and co-cultured with the supernatant of 4T1 cells treated with various treatments for 24 h. Subsequently, the BMDCs were co-cultured with lymphocytes for 48 h to activate T cells. The activated T cells were then co-cultured with 4T1 cells treated with various treatments for an additional 24 h. After incubation, the T cells were collected and stained with anti-CD3-FITC, anti-CD8-APC, and anti-CD107a-PE antibodies. The cytotoxic function of T cells, particularly the expression of the degranulation marker CD107a on CD8^+^ T cells, was assessed by flow cytometry to evaluate the cytotoxicity of T cells.

### 2.11 Evaluation of T cell killing efficiency

4T1 cells co-cultured with T cells were stained with crystal violet. The number of crystal violet-stained cells was observed under a microscope to assess the killing efficiency of T cells on 4T1 cells. A decrease in the number of crystal violet-stained cells indicated that the T cells had strong anti-tumor killing ability.

### 2.12 Research on cellular uptake of drugs

***Confocal Microscopy Observation:*** 4T1 cells were seeded at a density of 3 × 10^5^ per well in confocal dishes. After 24 h of incubation, the cells were subjected to the following two treatments: one part of the 4T1 cells was pre-treated with hyaluronic acid (10 mg/mL) for 4 h, followed by co-incubation with 3PNH (1 μg/mL) for 6 h. The other part of the 4T1 cells and COS7 cells were co-incubated with 3 PN (1 μg/mL) and 3PNH (1 μg/mL), respectively, for 6 h. After these treatments, all cells were stained with Hoechst 33342 (10 μg/mL) for 15 min and then observed for intracellular fluorescence distribution using CLSM.

***Flow Cytometry Quantitative Analysis:*** 4T1 cells were seeded at a density of 2 × 10^5^ per well in 12-well plates. Following the same treatment methods, the cells were washed three times with PBS. Subsequently, flow cytometry was employed to quantitatively analyze the cellular uptake, to assess the differences in uptake under various treatment conditions.

### 2.13 *in vivo* imaging

After successfully establishing a mouse breast cancer model, 3 PN or 3PNH (1 mg/mL, 200 μL) was injected intravenously into tumor-bearing mice. At time points of 0, 2, 4, 6, 8, 10, 12, and 24 h post-injection, the fluorescence distribution *in vivo* was monitored using an IVIS imaging system (Revvity). After 24 h, the major organs (heart, liver, spleen, lung, kidney) and tumor tissues were collected for ex vivo imaging analysis, to evaluate the *in vivo* distribution and metabolism of 3PNH.

### 2.14 *in vivo* antitumor and biosafety analysis

A 4T1 cell suspension (100 μL, cell density of 1.5 × 10^7^ cells/mL, containing 1.5 × 10^6^ cells) was subcutaneously injected into female Balb/C mice. When the tumor volume grew to approximately 300 mm^3^, the mice were randomly divided into the following groups: PBS control group, 3NPA group (15 mg/kg), 3PNH group (15 mg/kg), PH + L group (15 mg/kg), and 3PNH + L group (15 mg/kg). Every 3 days, the corresponding treatment were employed, and the mice of PH + L and 3PNH + L groups were irradiated with a laser (660 nm, 0.5 W/cm^2^, 5 min). The entire treatment lasted for 12 days. During the treatment, the body weight and tumor volume changes of the mice were regularly monitored. The tumor volume was calculated using the formula: tumor volume = length × width [2]/2. After the treatment, the mice were sacrificed, and the major organs (heart, liver, spleen, lung, kidney) and tumor tissues were collected. The weight of the tumor tissues was measured, and the excised tumor tissues were photographed. The resected tumor tissues and major organs were fixed in 4% paraformaldehyde, followed by hematoxylin-eosin (H&E) staining of pathological sections. Microscopic observation of the pathological changes in the tissues was conducted to evaluate the biosafety of the drugs. Mouse serum was collected and the levels of related inflammatory factors and biomarkers in the serum were analyzed using ELISA.

### 2.15 *in vivo* antitumor immune activation assessment

On the 13th day of treatment, after the mice were euthanized, tumor and spleen tissues were collected, washed with PBS, and prepared into single-cell suspensions, which were then washed and diluted to a cell concentration of 1 × 10^6^/mL.

***Spleen DCs Maturation Detection:*** the maturation of DCs in the spleen was detected using flow cytometry. Staining was performed with anti-CD11c-FITC, anti-CD80-PE/Cyanine5.5, and anti-CD86-APC antibodies. The maturation state of DC cells was assessed by detecting the expression levels of CD80 and CD86 on the surface of CD11c^+^ DC cells.

***Spleen T Cell Activation Detection:*** the activation of T cells in the spleen was detected using flow cytometry. Staining was performed with anti-CD3-FITC, anti-CD8-APC, and anti-CD69-PE antibodies. The activation state of T cells was assessed by detecting the expression level of CD69 on the surface of CD3^+^ T cells.

***T Cell Activation Detection in Tumor Tissue:*** The activation of T cells in the tumor tissue was detected using flow cytometry. Staining was performed with anti-CD3-FITC, anti-CD4-AF700, and anti-CD69-PE antibodies. The activation state of tumor-infiltrating T cells was assessed by detecting the expression level of CD69 on the surface of CD3^+^ T cells.

### 2.16 Validation of long-term immune memory *in vivo*

To establish an orthotopic breast cancer model, 1.5 × 10^6^ 4T1 cells were orthotopically injected into the left mammary fat pad of female Balb/C mice. When the tumor volume reached 100 mm^3^, the mice were randomly divided into five groups: PBS control group, 3NPA group (15 mg/kg), 3PNH group (15 mg/kg), PH + L group (15 mg/kg), and 3PNH + L group (15 mg/kg). The detailed treatment protocols for each group are described in Section 2.14. The treatment lasted for 13 days, after which the primary tumors in the left mammary fat pad were surgically excised, and the wounds were sutured. On day 16, 1.5 × 10^6^ 4T1 cells were re-injected into the right mammary fat pad of the mice to perform a tumor rechallenge experiment. Throughout the experiment, tumor volume was continuously monitored. On day 32, the mice were sacrificed, and spleen tissues were harvested for analysis. Flow cytometry was used to detect and analyze relevant immune cells within the spleen tissues.

### 2.17 Statistical analysis

Experimental data are presented as mean ± SD. Statistical analysis was performed using GraphPad Prism 10.0 software. One-way ANOVA followed by Tukey's test was used for statistical analysis. Differences with p < 0.05, p < 0.01, p < 0.001, and p < 0.0001 were considered statistically significant and are indicated with ∗, ∗∗, ∗∗∗, and ∗∗∗∗, respectively.

## 3 Results and discussion

### 3.1 Synthesis and characterization of 3PNH

**Fig. 1:**
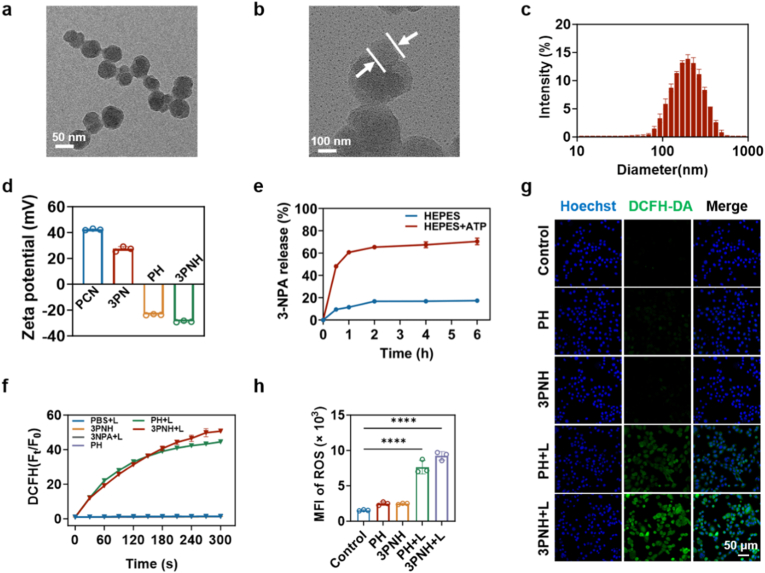
TEM images of (a) PCN-224 and (b) 3PNH. (c) The hydrodynamic size of 3PNH. (d) Zeta potentials of PCN, 3 PN, PH, and 3PNH (n = 3). (e) Release of 3NPA from 3PNH in HEPES buffer solutions containing different concentrations of ATP (n = 3). (f) ROS generation efficiency detected by a ROS detection kit under different conditions. (g) CLSM images of intracellular ROS generation in 4T1 cells under different treatment conditions detected by a ROS detection kit. (h) Analysis of intracellular ROS generation in 4T1 cells under different treatment conditions by flow cytometry (n = 3). Data are presented as mean ± SD. Statistical significance was calculated *via* one-way ANOVA with Tukey’ post hoc test. (∗p < 0.05, ∗∗p < 0.01, ∗∗∗p < 0.001, ∗∗∗∗p < 0.0001).

The synthesis of PCN-224 was carried out according to the methods described in references [39,40]. The morphology of PCN-224 was observed using transmission electron microscopy (TEM). As shown in Fig. 1a, PCN was found to be uniformly distributed and spherical in shape, with a size of approximately 70 nm. Similarly, 3PNH was observed to have the same structure and size as PCN, and a distinct core-shell structure was evident (Fig. 1b). The hydrodynamic size of 3PNH was measured by dynamic light scattering (DLS). With drug loading and HA modification, the hydrodynamic size of 3PNH was found to reach 177.8 nm (Fig. 1c). Upon HA modification, the zeta potential of 3PNH shifted from positive to negative (Fig. 1d). The stability of 3PNH was evaluated by comparing the changes in hydrodynamic particle size and zeta potential over 7 days in different solutions. As shown in Fig. S2, within 7 days, the hydrodynamic particle size and zeta potential of 3PNH remained essentially unchanged in both water and 10% fetal bovine serum (FBS), indicating that HA modification endows 3PNH with good stability. The UV absorption spectra of different nanoparticles were detected using a UV-Vis spectrophotometer. As shown in Fig. S3a, strong absorption peaks at 425 nm were exhibited by PCN, 3 PN, and 3PNH. Moreover, strong emission peaks at 680 nm under 440 nm excitation were also observed for all three nanoparticles. This indicates that neither the drug loading of 3NPA nor the surface modification with HA affected the optical properties of PCN (Fig. S3b). Furthermore, in the presence of 5 mg/mL of ATP, 72% of 3NPA was rapidly released from 3PNH. This is because the phosphate groups in ATP can strongly coordinate with metals, thereby disrupting the structure of PCN and causing rapid drug release (Fig. 1e).

DCFH-DA was used as a ROS probe to investigate the ability of 3PNH to generate ROS in solution. As shown in Fig. 1f, without light irradiation, the fluorescence intensity of the PH and 3PNH groups remained almost unchanged. However, after 300 s of light irradiation, the fluorescence intensity of these two groups increased nearly 45-fold, indicating that the ability of 3PNH to generate ROS under light stimulation was not affected. Correspondingly, the fluorescence intensity of the PBS and 3NPA groups remained almost unchanged under light irradiation, indicating that neither PBS nor 3NPA has the ability to generate ROS in solution. The ability of 3PNH to generate ROS intracellularly was also evaluated using DCFH-DA. As shown in Fig. 1g, no green fluorescence representing ROS production was observed in the Control, PH, and 3PNH groups. However, under light stimulation, obvious green fluorescence representing ROS production was exhibited by the PH + L group. In comparison, the 3PNH + L group showed brighter green fluorescence, indicating that 3PNH can generate more ROS within cells under light irradiation. This may be due to the release of 3NPA, which disrupts the mitochondrial electron transport chain function of tumor cells, thereby additionally generating a portion of mitochondrial ROS (mtROS). Furthermore, the production of intracellular ROS under different conditions was characterized by flow cytometry. As shown in Fig. 1h, more ROS was generated within cells in the 3PNH + L group. Additionally, a systematic evaluation of the hemolytic potential of 3PNH was conducted. As shown in Fig. S4, the hemolysis rate induced by 3PNH was extremely low (<2%) within the concentration range of 125-1000 μg/mL, comparable to that of the PBS negative control group. This finding clearly demonstrates the excellent *in vivo* biosafety of the 3PNH nanoplatform.

### 3.2 Investigation of the *in vitro* antitumor efficacy of 3PNH

**Fig. 2:**
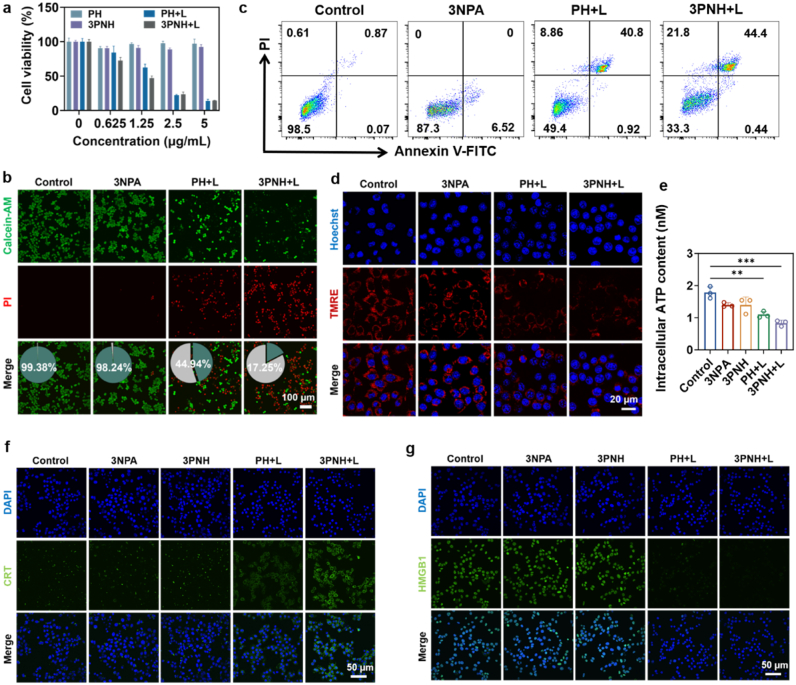
(a) Cell viability of 4T1 cells treated with PH and 3PNH, under light irradiation or in the dark. (“L” denotes light irradiation with a 660 nm laser at a power density of 0.5 W/cm^2^ for 5 min) (n = 4). (b) Cell viability of 4T1 cells assessed by staining with Calcein-AM/PI under different treatment conditions. (c) Detection of apoptosis in 4T1 cells by Annexin V-FITC/PI dual staining, analyzed *via* flow cytometry under different treatment conditions(n = 3). (d) Visualization of mitochondrial membrane potential in 4T1 cells by TMRE staining under different treatment conditions. (e) Intracellular ATP levels in 4T1 cells under different treatment conditions (n = 3). Immunofluorescence staining was used to evaluate (f) the expression of CRT in 4T1 cells and (g) the release of HMGB-1 in the nuclei of 4T1 cells under different treatment conditions. Data are presented as mean ± SD. Statistical significance was calculated *via* one-way ANOVA with Tukey’ post hoc test. (∗p < 0.05, ∗∗p < 0.01, ∗∗∗p < 0.001, ∗∗∗∗p < 0.0001).

The cytotoxicity of PH and 3PNH under different conditions was assessed using the CCK-8 assay. As shown in Fig. 2a, the cell viability of the PH and 3PNH groups was close to 100% in the absence of light stimulation, indicating their excellent biocompatibility in the dark. However, cytotoxicity was significantly enhanced when light stimulation was introduced. Specifically, at a PCN concentration of 1.25 μg/mL, the cell viability of the PH + L and 3PNH + L groups decreased to approximately 50%. This result is consistent with the data in Fig. 1g and h, where the cell viability of the 3PNH + L group was slightly lower than that of the PH + L group under the same concentration conditions. This further confirms that more ROS can be generated intracellularly by 3PNH under light stimulation, leading to a higher cell death rate. In addition, the phototoxicity of PCN-224 before and after HA decoration was further quantified. Under identical PCN-equivalent doses and light irradiation, HA-modified PCN-224 (PH) evoked significantly lower cell viability than its bare counterpart (Fig. S5), demonstrating that HA conjugation potentiates the photodynamic efficacy of PCN-224. This enhancement is ascribed to CD44-mediated active internalization, which elevates intracellular photosensitizer accumulation and thereby amplifies the ROS-triggered cytotoxicity within tumor cells. The viability and mortality of 4T1 cells under different conditions were further verified using a live/dead cell staining assay. As shown in Fig. 2b, almost no red fluorescence representing cell death was observed in the Control and 3NPA groups, while distinct red fluorescence was observed in the PH + L and 3PNH + L groups, indicating a large number of dead cells in these groups. Based on the counts and statistical analysis of cells in multiple fields of view using image analysis software, the cell viability of 3PNH + L group was calculated to be approximately 17.25% and the mortality rate was approximately 82.75% (Fig. S6). The *in vitro* antitumor efficacy of 3PNH was evaluated using a 3D cell spheroid model. As shown in Fig. S7a, after 6 days of treatment, the tumor cell spheroid volume in the 3PNH + L group was significantly reduced, with a greater reduction than that observed in the PH + L group (Fig. S7b). This result indicates that the antitumor treatment strategy combining 3NPA with PDT has a significantly stronger inhibitory effect on tumor cell growth than PDT by itself.

The apoptosis of cells under different treatment conditions was next detected using an apoptosis and necrosis kit. As shown in Fig. 2c, compared with the control group, 6.52% of 4T1 cells treated with 3NPA entered the early apoptosis stage. This result further confirmed that interference with the mitochondrial electron transport chain by 3NPA leads to an increase in intracellular ROS levels, thereby activating the apoptosis pathway. The proportion of PI-positive cells representing late apoptosis reached 44.4% in the 3PNH + L group and 40.8% in the PH + L group (Fig. S8). These data strongly support the conclusion shown in Fig. 2b, indicating that the relevant treatments can significantly induce apoptosis and thereby promote cell death. Next, the mitochondrial membrane potential of 4T1 cells under different conditions was characterized using the TMRE probe, and the cells were observed *via* confocal laser scanning microscopy (CLSM). As shown in Fig. 2d, bright red fluorescence was exhibited by the mitochondria of 4T1 cells in the Control group, indicating that the mitochondrial membrane potential was at a normal level. In contrast, after light treatment, a significant reduction in the fluorescence intensity of the mitochondria was observed in the PH + L and 3PNH + L groups of 4T1 cells, indicating a decrease in mitochondrial membrane potential in these two groups. This suggests that the depolarization of the mitochondrial membrane potential led to the release of TMRE from the mitochondria. Quantitative analysis of the images was performed using ImageJ software. As shown in Fig. S9, the average fluorescence intensity of the 3PNH + L group was significantly lower than that of the Control group. Notably, the average fluorescence intensity of the 3NPA group was also slightly lower than that of the Control group. This may be related to the inhibition of mitochondrial CII by 3NPA, which subsequently leads to mitochondrial dysfunction in 4T1 cells. Moreover, the decrease in mitochondrial membrane potential may also be associated with early events of apoptosis, as depolarization of the mitochondrial membrane potential is a key hallmark of apoptosis.

To further investigate the ability of 3PNH to induce ICD in tumor cells under light stimulation, the secretion of ATP, the expression of CRT, and the release of HMGB1 in tumor cells under different treatment conditions were examined. As shown in Fig. 2e, the intracellular ATP content in 4T1 cells treated with 3PNH + L was significantly lower compared to the Control group, indicating that treatment with 3PNH + L markedly promoted the extracellular secretion of ATP by 4T1 cells. The expression of CRT and the release of HMGB1 in 4T1 cells after different treatments were further localized using immunofluorescence staining. As shown in Fig. 2f and g, increased CRT expression on the cell surface and more complete release of HMGB1 from the nucleus were observed in 4T1 cells treated with 3PNH + L. The quantitative fluorescence intensity results were more intuitively presented in Fig. S10. These findings indicate that treatment with 3PNH under light irradiation significantly induces ICD in 4T1 cells, resulting in the release of tumor antigens and the activation of downstream immune responses.

### 3.3 3PNH is capable of activating DCs

**Fig. 3:**
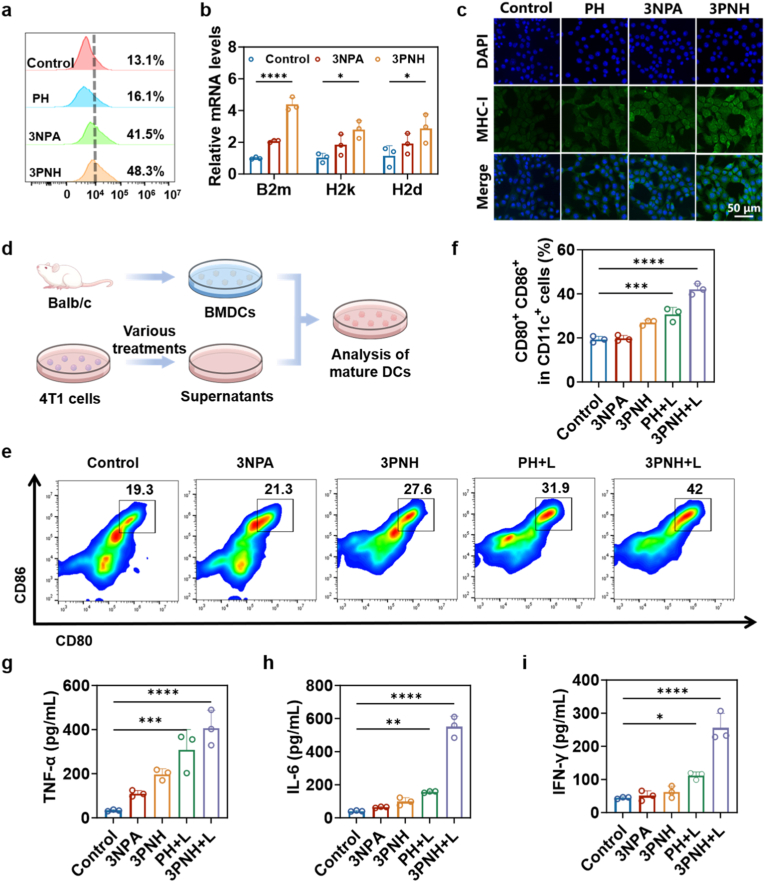
(a) Flow cytometric quantification of MHC-I expression in 4T1 cells following 24-h treatment with PH, 3NPA, or 3PNH. (b) RT-qPCR analysis of MHC-I associated gene expression in 4T1 cells treated with 3NPA and 3PNH(n = 3). (c) The expression of MHC-I protein in 4T1 cells under different conditions was evaluated by immunofluorescence staining and observed using CLSM. (d) Schematic illustration of the experimental design to evaluate the activation of DCs. (e) Flow cytometric determination of the proportion of mature DCs *in vitro*, and (f) the corresponding quantitative analysis (n = 3). (g)–(i) Quantitative analysis of cytokine secretion (TNF-α, IL-6, IFN-γ) from DCs measured by ELISA (n = 3). Data are presented as mean ± SD. Statistical significance was calculated *via* one-way ANOVA with Tukey’ post hoc test. (∗p < 0.05, ∗∗p < 0.01, ∗∗∗p < 0.001, ∗∗∗∗p < 0.0001).

The regulatory effects of 3NPA on the expression levels of MHC-I protein in 4T1 tumor cells were further investigated using flow cytometry. As clearly shown in Fig. 3a, compared with the Control group, no significant change was observed in the expression of MHC-I protein in tumor cells of the PH group. However, in tumor cells treated with 3NPA or 3PNH, the expression of MHC-I protein was significantly increased to 41.5% and 48.3%, respectively. These results strongly confirmed that 3NPA could significantly upregulate the expression of MHC-I protein in tumor cells by inhibiting the activity of mitochondrial electron transport chain CII. Notably, under the same 3NPA concentration (30 μg/mL), the increase in MHC-I protein expression in tumor cells treated with 3PNH was higher than that in the 3NPA group (48.3% vs. 41.5%). This was likely due to the unique nanostructure of 3PNH and the targeting modification of HA, which enhanced the endocytosis of the drug by tumor cells, thereby resulting in a more significant expression of MHC-I protein in tumor cells treated with 3PNH. According to recent work [38], 3NPA-mediated inhibition of CII raises intracellular succinate, lowers the α-KG/succinate ratio, and suppresses KDM4/5 histone demethylases. The resulting enrichment of H3K4me3 and H3K36me3 at MHC-APP loci boosts NLRC5-dependent transcription, thereby increasing MHC-I expression independently of IFN-γ. Guided by this mechanism, RT-qPCR was employed to quantify MHC-APP gene expression in 4T1 cells under the indicated treatments. As shown in Fig. 3b, compared with the Control group, the relative mRNA levels of MHC-APP related genes (B2m, H2k, H2d) in 4T1 cells treated with 3NPA or 3PNH were significantly increased. This further strongly indicated that 3NPA could effectively promote the expression of MHC-APP related genes in 4T1 cells, thereby driving the expression of MHC-I protein. Consistent with the results in Fig. 3a, the expression of MHC-APP related genes in the 3PNH group was more significant than that in the 3NPA group. This further confirmed that, compared with free 3NPA, 3PNH, after being modified with HA and loaded with PCN, had a stronger ability to efficiently deliver 3NPA into 4T1 cells, thus showing a more significant effect in promoting the expression of MHC-I protein. Additionally, the ability of 3NPA and 3PNH to promote the expression of MHC-I protein was further confirmed through immunofluorescence staining. The expression trend of MHC-I protein, as shown in Fig. 3c and supported by the semi-quantitative analysis in Fig. S12, was found to be highly consistent with that illustrated in Fig. 3a. These observations further substantiate the ability of 3NPA to significantly enhance MHC-I expression in tumor cells.

Subsequently, the activation status of DCs under various treatment conditions was comprehensively evaluated using flow cytometry. As shown in Fig. 3d, mouse bone marrow cells were first collected and induced to differentiate into bone marrow-derived dendritic cells (BMDCs). The BMDCs were then cultured with tumor cell culture supernatants under different treatment conditions, and the expression of DC activation markers was analyzed using flow cytometry. As shown in Fig. 3e and f, compared with the Control and 3NPA groups, the supernatant of tumor cells treated with 3PNH significantly stimulated the maturation of DCs. This result is highly consistent with the findings presented in Fig. 3a and b, further confirming that the tumor-conditioned medium (TCM) from the 3PNH group exhibits a significantly greater capacity to promote the maturation of DCs compared to that from the 3NPA group. Specifically, the TCM from the 3PNH group enabled 27.6% of DCs to reach a mature state. As is well known, PDT can induce tumor cells to undergo ICD [10,11] releasing a large amount of tumor antigens, thereby stimulating the maturation of DCs. In this experiment, the proportion of mature DCs in the TCM from the PH + L group was 31.9%, while the TCM from the 3PNH + L group, by synergizing PDT with the function of 3NPA, achieved a DC maturation rate of 42%. This increase in proportion highlights the unique advantage of the TCM from the 3PNH + L group in promoting DC maturation. In terms of cytokine expression in the supernatant of DCs, the DCs stimulated by the TCM from the 3PNH + L group released more TNF-α and IL-6 (Fig. 3g and h).

This phenomenon indicates that the DCs stimulated by the TCM from 3PNH + L group are in an activated and pro-inflammatory state, capable of effectively triggering downstream cascade immune responses. Moreover, compared with other groups, the concentration of IFN-γ secreted by the 3PNH + L group was significantly increased (Fig. 3i). As a key immune-regulatory cytokine, the increase in IFN-γ concentration can significantly enhance the antigen-presenting capacity of DCs, promote the expression of MHC molecules and co-stimulatory molecules on the surface of DCs, thereby enabling DCs to more efficiently activate T cells, especially CD8^+^ T cells. This enhancement is of great significance for activating specific immune responses and strengthening the body's immune surveillance and killing ability against tumor cells.

### 3.4 3PNH significantly augments the cytotoxic activity of CD8^+^ T cells towards tumor cells

**Fig. 4:**
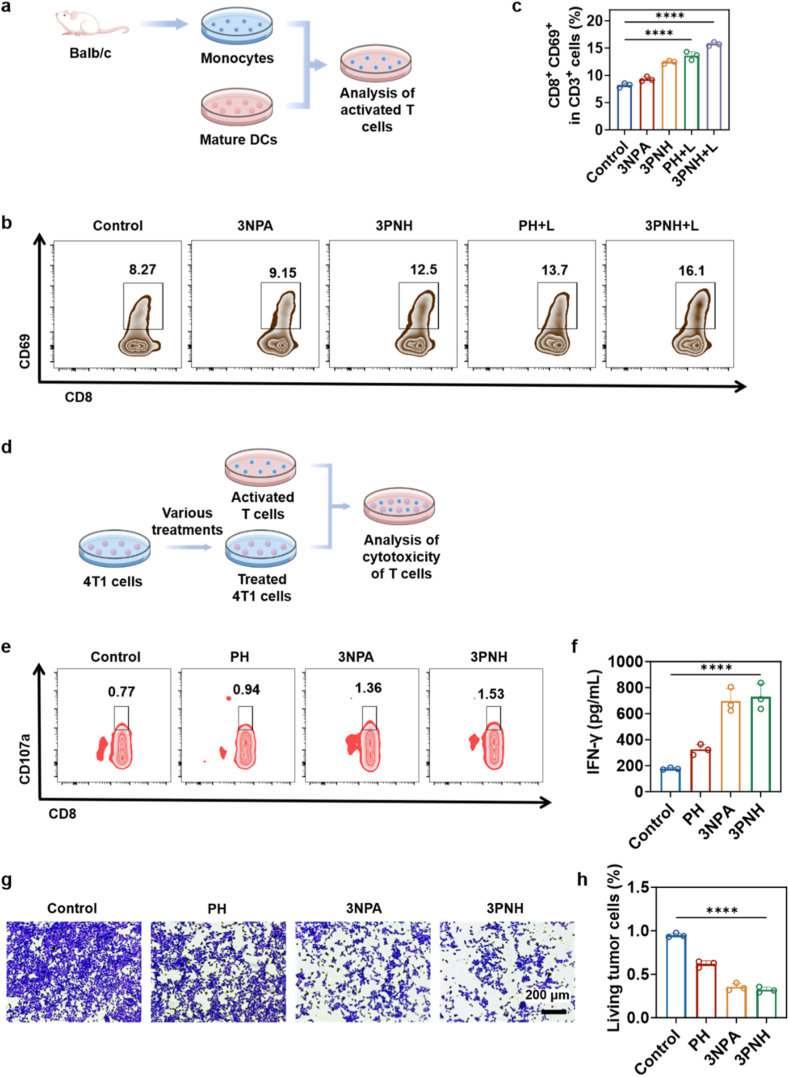
(a) Schematic illustration of the experimental design to evaluate T cells activation. (b) Flow cytometric detection of activated T cells (CD3^+^CD8^+^CD69^+^) *in vitro*, and (c) corresponding quantitative analysis (n = 3). (d) Schematic illustration of the experimental design to evaluate the cytotoxicity of activated T cells. (e) Flow cytometric analysis of cytotoxicity-related markers (CD3^+^CD8^+^CD107a^+^) on activated T cells. (f) Quantification of IFN-γ secretion from activated T cells by ELISA (n = 3). (g) Assessment of the cytotoxic effect of activated T cells on 4T1 cells using a colony formation assay, and (h) related quantitative analysis (n = 3). Data are presented as mean ± SD. Statistical significance was calculated *via* one-way ANOVA with Tukey’ post hoc test. (∗p < 0.05, ∗∗p < 0.01, ∗∗∗p < 0.001, ∗∗∗∗p < 0.0001).

Furthermore, as shown in Fig. 4a, mature DCs obtained under different conditions (as shown in Fig. 3e and f) were co-cultured with lymphocytes isolated from mouse spleens, and the expression of T cell activation markers was precisely analyzed using flow cytometry. As shown in Fig. 4b and c, compared with the other four groups, a significantly higher proportion (16.1%) of lymphocytes expressing the maturation-related protein CD3^+^CD8^+^CD69^+^ was observed in the co-culture system of DCs stimulated by the TCM from 3PNH + L group. This indicates that the TCM from 3PNH + L group significantly enhanced the ability of DCs to stimulate lymphocytes, enabling them to more effectively induce lymphocyte maturation. This process further modulates the activation and functional status of immune cells, providing robust support for subsequent immune responses.

Subsequently, a series of experiments were conducted to investigate whether treatment with 3NPA would affect the susceptibility of tumor cells to T cell-mediated cytotoxicity by altering the expression of MHC-I proteins. As shown in Fig. 4d, T1 cells subjected to different pretreatments were co-cultured with lymphocytes activated by the 3PNH + L group (as shown in Fig. 4b). The cytotoxicity of T cells against tumor cells under various treatment conditions was systematically assessed using flow cytometry and crystal violet assays. Meanwhile, the levels of multiple cytokines in the supernatants of the co-culture systems were detected by ELISA assays, thereby providing a comprehensive evaluation of T cell cytotoxicity under different treatment conditions. As shown in Fig. 4e and S14, compared with the control group, no significant changes in the expression levels of the cytotoxicity-related marker protein CD107a were observed in the co-cultured cells treated with PH. However, a significant upregulation of CD107a was observed in the co-cultured cells treated with 3NPA and 3PNH, with this upregulation being more pronounced than in the PH group. These results are highly consistent with the MHC-I protein expression patterns observed in Fig. 3a and b, further indicating that both 3NPA and 3PNH significantly enhance the expression of MHC-I protein in 4T1 cells, with 3PNH inducing higher levels of MHC-I protein expression than 3NPA. Therefore, 3PNH more effectively increases the proportion of CD3^+^CD8^+^CD107a^+^ T cells. Integrating the results from Figs. 4e, 3a and 3b, it can be clearly concluded that the upregulation of MHC-I protein expression in 4T1 cells significantly enhances the ability of T lymphocytes to recognize and kill tumor cells. The increased expression of MHC-I protein provides more effective target signals for T cells, thereby significantly enhancing their cytotoxic function against tumor cells. Additionally, the expression levels of the cytokine IFN-γ, which is closely associated with T lymphocyte cytotoxicity, were measured in the supernatants of the co-cultured system. As shown in Fig. 4f, the expression levels of IFN-γ in the supernatants of co-cultured system from the 3NPA and 3PNH groups were significantly higher. Moreover, crystal violet staining was performed on the co-cultured system to more intuitively assess the cytotoxicity of T cells. As shown in Fig. 4g and h, fewer purple-stained cells, representing surviving tumor cells, were observed in the 3NPA and 3PNH groups, indicating a significant enhancement in T lymphocyte cytotoxicity in these groups. These results further confirm that the upregulation of MHC-I protein expression significantly enhances the cytotoxic function of T cells.

### 3.5 *in vitro* tumor-targeting efficacy and *in vivo* biodistribution of 3PNH

**Fig. 5:**
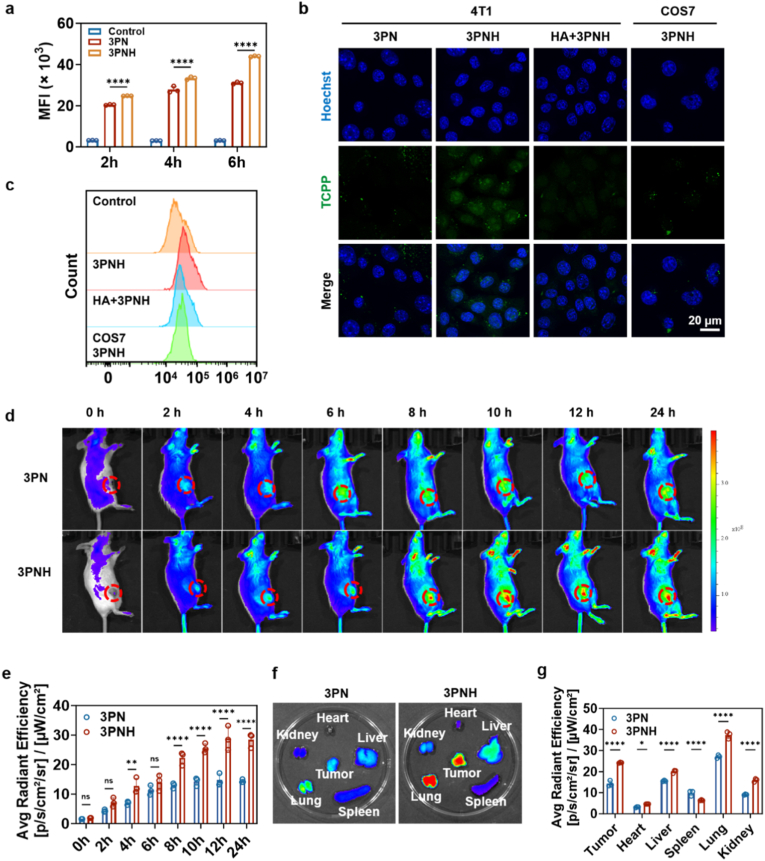
(a) Cellular uptake levels of 3 PN and 3PNH in 4T1 cells at different incubation times, as determined by flow cytometry (n = 3). (b) Targeting effects of HA on 4T1 cells under various conditions, evaluated by CLSM and (c) flow cytometric analysis. (d) *In vivo* fluorescence imaging of 4T1 tumor-bearing mice following intravenous injection of 3 PN and 3PNH. (e) Semi-quantitative analysis of fluorescence intensity in the tumor region at different indicated time points (n = 3). (f) Biodistribution imaging of major organs at 24 h post-injection, and (g) semi-quantitative analysis (n = 3). Data are presented as mean ± SD. Statistical significance was calculated *via* one-way ANOVA with Tukey’ post hoc test. (∗p < 0.05, ∗∗p < 0.01, ∗∗∗p < 0.001, ∗∗∗∗p < 0.0001).

The tumor-targeting ability of 3PNH was investigated in the following section. The ability of 3 PN and 3PNH to enter 4T1 cells was compared using flow cytometry. As shown in Fig. 5a, under the same incubation time conditions, 3PNH was found to enter 4T1 cells to a greater extent than 3 PN, indicating that modification with HA enhanced the targeting ability of 3PNH towards 4T1 cells. The CD44-targeting ability of 3PNH was also observed using CLSM. As depicted in Figs. 5b and 4T1 cells were pre-treated with free HA to block the CD44 protein on the cell surface. The results showed that, compared with the 3PNH group, the red fluorescence representing 3PNH in 4T1 cells was significantly weaker after pre-treatment with HA. Additionally, COS7 cells with low CD44 expression were used as a control. The fluorescence intensity in 4T1 cells was found to be significantly stronger than that in COS7 cells after incubation with 3PNH. The analysis of cellular fluorescence intensity by flow cytometry more intuitively illustrates the aforementioned results (Fig. 5c).

The *in vivo* tumor-targeting ability of 3PNH was further evaluated by intravenous injection of 3 PN and 3PNH. As shown in Fig. 5d, significant drug accumulation was observed in the tumor region (indicated by the red circle) in the 3PNH group 2 h after injection, and this accumulation increased gradually over time. The fluorescence in the tumor region remained very strong, even 24 h after injection. It is known that the three-dimensional porous nanostructure of 3 PN can achieve relatively good accumulation in tumor tissues through the enhanced permeability and retention (EPR) effect [41,42]. However, compared with the 3 PN group, better targeting and accumulation effects were observed for 3PNH at the same time point (Fig. 5e). After intravenous injection for 24 h, the major organs (heart, liver, spleen, lung, and kidney) and tumor tissues were harvested from the mice and subjected to fluorescence imaging. As shown in Fig. 5f and g, stronger fluorescence intensity was observed in the tumor tissue of the 3PNH group compared with the 3 PN group. This further demonstrates the importance of HA modification in enhancing the tumor-targeting ability of 3PNH and also confirms the good retention effect of 3PNH in the tumor tissue after targeting.

### 3.6 Investigation of the *in vivo* antitumor efficacy of 3PNH

**Fig. 6:**
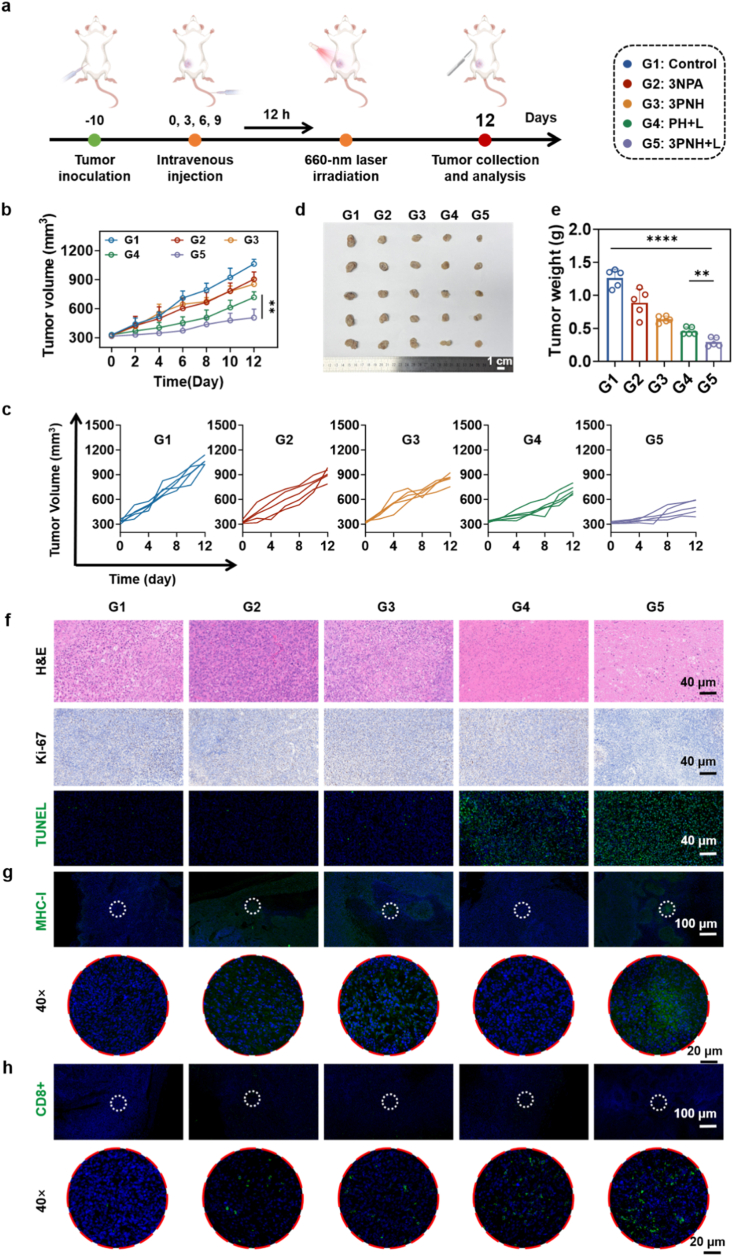
(a) Schematic illustration of the *in vivo* antitumor treatment process over 12 days. (b) Tumor growth curves of different treatment groups (n = 5) and (c) individual mouse tumor growth profiles (n = 5). (d) Representative photographs of excised tumor tissues after treatment and (e) corresponding tumor weight measurements (n = 5). (f) H&E, Ki-67, and TUNEL staining of tumor tissues after treatment. (g) Immunofluorescence staining of MHC-I and (h) CD8^+^ T cells in tumor tissues at different magnifications after treatment. Data are presented as mean ± SD. Statistical significance was calculated *via* one-way ANOVA with Tukey’ post hoc test. (∗p < 0.05, ∗∗p < 0.01, ∗∗∗p < 0.001, ∗∗∗∗p < 0.0001).

The *in vivo* anti-tumor efficacy of 3PNH was systematically evaluated using a mouse TNBC model. As shown in Fig. 6a, different drugs were administered *via* intravenous injection on days 0, 3, 6, and 9 of the treatment. Based on the results in Fig. 5e, light irradiation was applied to the tumors in the mice 12 h postinjection, and the mice were euthanized on day 12 of the treatment. As shown in Fig. 6b and c, the tumor inhibition effect in the 3PNH + L group was the most significant throughout the 12-day treatment period compared with the other four groups. The image of tumor tissues in Fig. 6d and the tumor weight data in Fig. 6e more intuitively demonstrated the anti-tumor advantages of the 3PNH + L group. Fig. S15 shows that the body weights of mice in all groups did not change significantly throughout the treatment process, indicating that 3NPA and 3PNH did not cause noticeable systemic toxicity at the appropriate dosing concentrations. After the treatment, H&E staining was performed on the major organs of the mice, and the results shown in Fig. S16 revealed no significant pathological changes in the major organs during the treatment.

The tumor tissues from each group of mice were subjected to in-depth analyses. As shown in Fig. 6f–H&E staining revealed distinct necrotic and apoptotic areas in the tumor tissues of the 3PNH + L group. The Ki-67 staining results indicated that the tumor inhibition effect in the 3PNH + L group was the most outstanding among the five groups. The TUNEL staining results clearly showed a large amount of green fluorescence representing cell apoptosis in the 3PNH + L group. These results further confirmed that the 3PNH + L treatment strategy can significantly inhibit tumor cell growth and enhance therapeutic efficacy. To further investigate the impact of the 3PNH + L treatment strategy on the tumor immune microenvironment, immunofluorescence staining was used to analyze the expression of relevant proteins in the tumor tissues of each group. As shown in Fig. 6g, the expression level of MHC-I protein was significantly increased in the 3PNH + L group. Meanwhile, Fig. 6h showed that the infiltration level of CD8^+^ T lymphocytes in the tumor tissues of the 3PNH+L group was also significantly enhanced. Collectively, these results indicate that the 3PNH + L treatment strategy stimulates tumor cells to upregulate MHC-I protein expression, enhances the antigen-presenting capacity of DCs, and thereby promotes the infiltration and activation of CD8^+^ T cells.

### 3.7 Investigation of the immune-activating efficacy of 3PNH *in vivo*

**Fig. 7:**
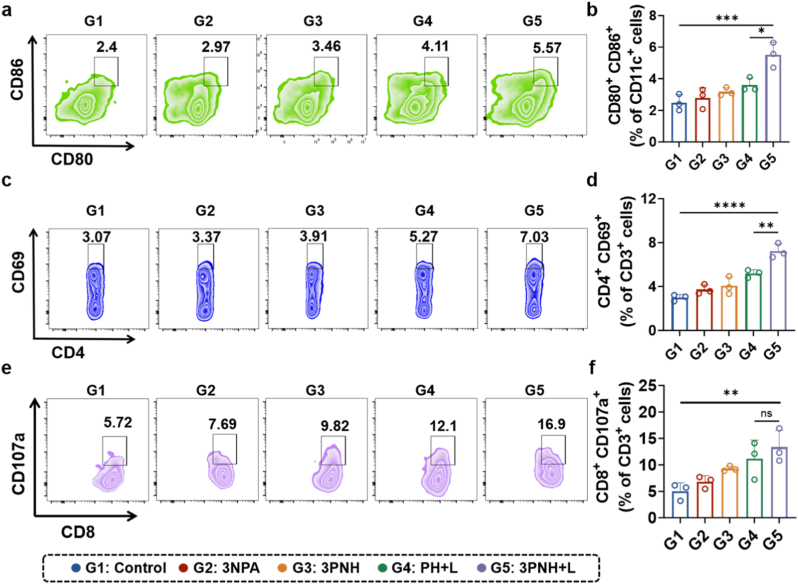
Flow cytometric phenotyping of tumor-infiltrating immune cells, including (a) mature DCs (identified by CD11c^+^CD80^+^CD86^+^). (c) activated CD4^+^ T cells (identified by CD3^+^CD4^+^CD69^+^). (e) cytotoxic T lymphocytes (identified by CD3^+^CD8^+^CD107a^+^). Additionally, quantitative assessment of the aforementioned immune cell subsets is presented in (b), (d), and (f), respectively (n = 5). Data are presented as mean ± SD. Statistical significance was calculated *via* one-way ANOVA with Tukey’ post hoc test. (∗p < 0.05, ∗∗p < 0.01, ∗∗∗p < 0.001, ∗∗∗∗p < 0.0001).

Furthermore, the proportion of mature DCs in the tumor tissues was analyzed using flow cytometry after tissue homogenization and preparation of single-cell suspensions. As shown in Fig. 7a and b, compared with the other four groups, the proportion of mature DCs in the tumor tissues of mice treated with 3PNH + L was significantly higher. This suggests that the 3PNH + L treatment strategy effectively promoted the maturation and activation of DCs. Consequently, the enhanced function of DCs likely facilitated their subsequent more efficient uptake, processing, and presentation of antigens, thereby activating T lymphocytes. The activation of helper T cells in the tumor tissue was characterized using CD3^+^CD4^+^CD69^+^ markers. As shown in Fig. 7c and d, compared with the other four groups, the proportion of activated helper T cells in the tumor tissue of the 3PNH + L group was the highest. Given that the activation of helper T cells largely determines the immune surveillance of the body against tumors and the proliferation and maturation of cytotoxic T lymphocytes, this further demonstrates that the treatment strategy of PDT combined with 3NPA can significantly enhance the activity of immune cells in the tumor tissue and improve the recognition and killing of tumor cells by cytotoxic T cells. Subsequently, the cytotoxic function of CD8^+^ T cells in the tumor tissues was validated using CD3^+^CD8^+^CD107a^+^ markers. As illustrated in Fig. 7e and f, compared with the Control group, a significant increase was observed in the proportion of CD8^+^ T cells exhibiting enhanced cytotoxicity in the tumor tissues of mice treated with 3NPA and 3PNH. Notably, the greatest increase was observed in the 3PNH + L group, with the proportion rising from 5.72% to 16.9%. This suggests that the strategy combining PDT with 3NPA can significantly enhance the cytotoxicity of CD8^+^ T cells infiltrating the tumor tissues, enabling them to release more perforin and granzymes, thereby more effectively inducing tumor cell apoptosis.

**Fig. 8:**
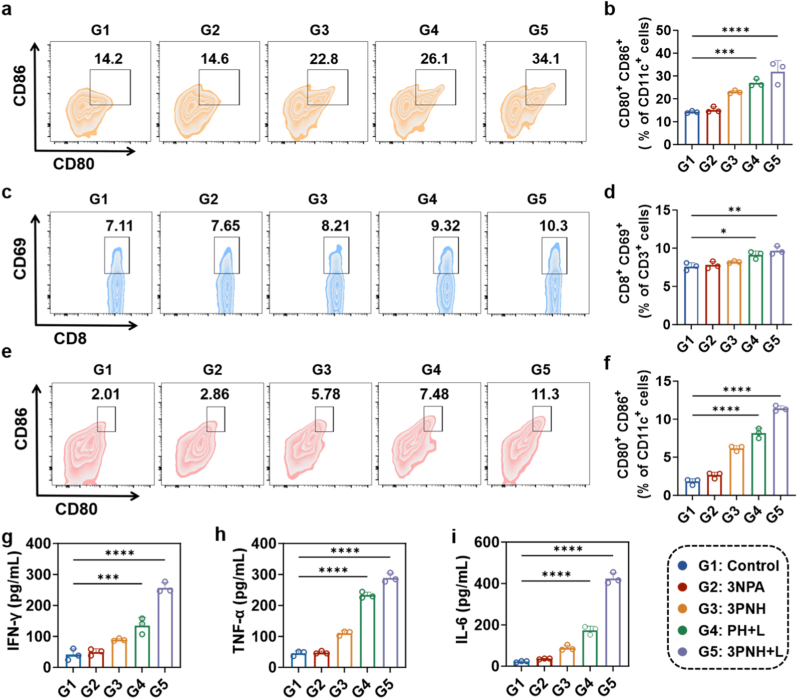
(a) Flow cytometric analysis of mature DCs, identified by CD11c^+^CD80^+^CD86^+^ and (c) activated CD8^+^ T cells, identified by CD3^+^CD8^+^CD69^+^ in the spleens of mice after treatment, with quantitative results shown in (b) and (d), respectively. (e) Flow cytometric analysis of mature DCs in the lymph nodes of mice, and (f) the corresponding quantitative results. Concentrations of cytokines in mouse serum after treatment (g) IFN-γ, (h) TNF-α, and (i) IL-6 (n = 5). Data are presented as mean ± SD. Statistical significance was calculated *via* one-way ANOVA with Tukey’ post hoc test. (∗p < 0.05, ∗∗p < 0.01, ∗∗∗p < 0.001, ∗∗∗∗p < 0.0001).

The *in vivo* immune activation induced by 3PNH was evaluated using flow cytometry. The proportion of immune cells in the spleen tissues of mice was initially examined after 12 days of treatment. As shown in Fig. 8a and b, the proportion of mature DC cells in the spleen tissues was characterized using CD11c^+^CD80^+^CD86^+^ markers. Compared to the Control group, the proportion of activated DC cells in the 3PNH group increased from 14.2% to 22.8%, while no significant change was observed in the 3NPA group. This indicates that the role of PCN as a drug carrier is significant in *in vivo* drug delivery, effectively enhancing the delivery efficiency of 3NPA and thereby boosting the immune response. Compared with the other three groups, the proportion of CD11c^+^CD80^+^CD86^+^ cells in the PH+L and 3PNH + L groups was significantly increased, rising to 26.1% and 34.1%, respectively. This demonstrates that PDT plays a crucial role in increasing the proportion of mature DC cells in the spleen tissue. The activation state of CD8^+^ T cells in the spleen tissue was characterized using CD3^+^CD8^+^CD69^+^ markers. As shown in Fig. 8c and d, the proportion of CD3^+^CD8^+^CD69^+^ cells in the spleen tissues of mice in the PH+L and 3PNH + L groups was higher than that in the other three groups. This further confirms that PDT, as well as the combination of PDT and 3NPA, can effectively activate CD8^+^ T cells in the spleen, enhancing their immune activity and thereby exerting antitumor immune effects.

The proportion of activated DCs in the mouse lymph nodes, marked by CD11c^+^CD80^+^CD86^+^ cells, was further examined. As shown in Fig. 8e and f, compared with the Control group, a significant increase was observed in the proportion of activated DCs in the lymph nodes of the 3PNH group, PH + L group, and 3PNH + L group. Among these groups, the proportion of activated DCs in the 3PNH + L group was the most prominent, reaching 11.3%. This indicates that the 3PNH + L treatment strategy can significantly activate the immune response of the body. Mature DCs in the lymph nodes play a key role in processing and presenting tumor antigens, thereby laying a solid foundation for the subsequent specific anti-tumor immune response. In addition, the concentrations of IFN-γ, TNF-α, and IL-6 in the mouse serum were measured after the end of treatment. As shown in Fig. 8g, h, and 8i, a significant increase in the concentration of IFN-γ was observed in the serum of mice in the 3PNH + L group, which further validates the findings in Fig. 8e, that is, the activated DCs in the lymph nodes can effectively stimulate T lymphocytes to secrete more IFN-γ. Meanwhile, the concentrations of TNF-α and IL-6 in the serum of mice in the 3PNH + L group were also significantly elevated, reflecting the inflammatory response accompanying immune activation. These results suggest that the 3PNH + L treatment regimen not only activates immune cells but also enhances the overall immune response of the body through the synergistic action of multiple cytokines, providing strong support for anti-tumor therapy.

**Fig. 9:**
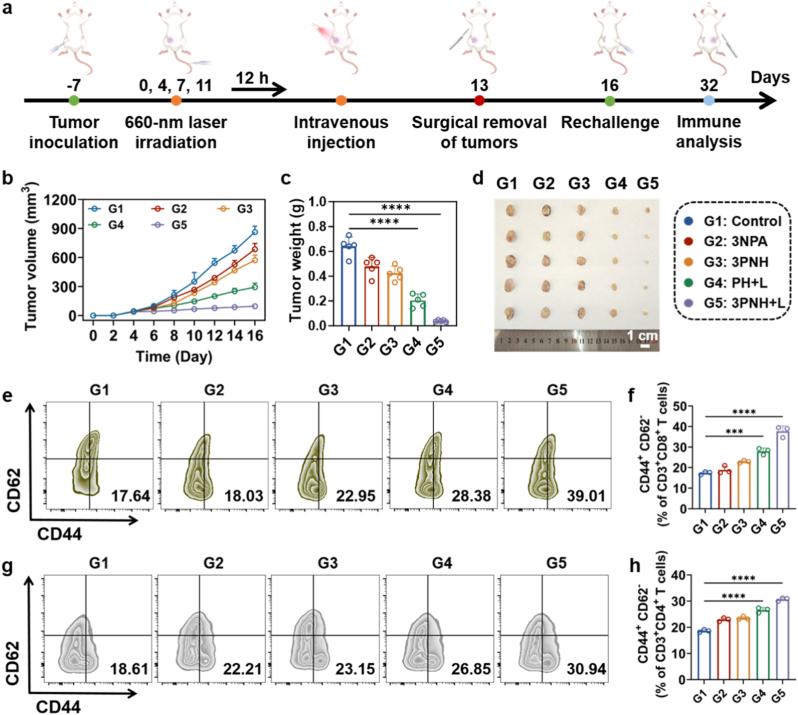
(a) Schematic diagram of the *in vivo* tumor rechallenge experiment. (b) Tumor growth curve over a 16-day observation period following the re-injection of tumor cells on day 16. (c) Weight of the re-grown tumors after completion of the tumor rechallenge experiment and (d) Photographs of the re-challenged tumor tissues excised on day 32 after re-inoculation of tumor cells on day 16. After the completion of the tumor rechallenge experiment, the spleen tissues from mice in each group were analyzed by flow cytometry to examine (e) CD8^+^ effector memory T cells and (g) CD4^+^ effector memory T cells, with corresponding quantitative results shown in (f) and (h). Data are presented as mean ± SD. Statistical significance was calculated *via* one-way ANOVA with Tukey’ post hoc test. (∗p < 0.05, ∗∗p < 0.01, ∗∗∗p < 0.001, ∗∗∗∗p < 0.0001).

To systematically evaluate whether 3PNH-mediated immunotherapy can induce long-lasting antitumor immune memory, we performed surgical resection of the primary tumor on day 13. Subsequently, on day 16, an equal number of 4T1 cells were re-inoculated into the contralateral mammary fat pad to establish a tumor rechallenge model (Fig. 9a). During the 16-day observation period, the tumor growth rate in Group G4 was significantly reduced compared to G1-G3 groups, suggesting that the ICD induced by PH can partially activate immune memory and confer inhibitory capacity against reinvading tumor cells (Fig. 9b). Notably, Group G5 exhibited a more remarkable tumor suppression effect, with tumor growth being almost completely inhibited (Fig. 9b–d). These results strongly demonstrate that the combination of PDT and enhanced MHC-I expression integrated in 3PNH can synergistically drive a more thorough and long-lasting antitumor immune memory.

To elucidate the immunological characteristics of this immune memory at the cellular level, spleens from all groups were harvested at the endpoint of the rechallenge experiment on day 32 and analyzed for effector memory T cell (TEM) populations using multiparameter flow cytometry. As shown in Fig. 9e and f**,** the proportion of CD8^+^ TEM cells (CD3^+^CD8^+^CD62L^−^CD44^+^) significantly increased from 17.64% in Group G1 to 39.01% in Group G5, representing a 121.3% increase. This pattern of change closely correlates with the robust tumor-suppressive function observed in the 3PNH + L group, confirming the presence of a highly effective antitumor T cell reservoir *in vivo*. Concurrently, CD4^+^ TEM cells (CD3^+^CD4^+^CD62L^−^CD44^+^) also exhibited a synergistic increase, rising from 18.61% in Group G1 to 30.94% in Group G5 (a 66.3% increase, Fig. 9g and h). Although this increase was less pronounced than that of CD8^+^ TEM, it still indicated that CD4^+^ helper memory cells were synchronously activated, likely providing survival and functional support to CD8^+^ TEM through the secretion of key factors such as IL-2. Statistical analysis further revealed that both TEM subsets in Group G5 were significantly higher than those in Group G1 (p < 0.001), and the relative increase of CD8^+^ TEM was significantly greater than that of CD4^+^ TEM, highlighting the dominant role of cytotoxic responses in 3PNH-mediated immune memory.

## 4 Conclusions

A novel nanoplatform, 3PNH, was successfully developed in this study, offering an innovative strategy for the efficient treatment of low-immunogenic TNBC. The therapeutic efficacy against TNBC was significantly enhanced by the 3PNH through the synergistic induction of ICD and the upregulation of MHC-I expression. Specifically, ICD was effectively induced by the photosensitizer PCN-224 *via* PDT, which prompted the release of DAMPs from tumor cells and thereby activated the immune response. Meanwhile, the sustained release of 3NPA inhibited the activity of mitochondrial CII in tumor cells, leading to the upregulation of MHC-I expression. This alteration not only significantly enhanced the antigen-presenting capacity of DCs but also markedly improved the recognition and killing efficiency of CD8^+^ T cells against tumor cells. Overall, low-immunogenic TNBC was successfully transformed into a more immunogenic “hot tumor” by the 3PNH, paving a new way for antitumor immunotherapy. This innovative strategy not only provides a novel approach for the clinical treatment of TNBC but also offers a potential idea for the treatment of other low-immunogenic tumors.

## CRediT authorship contribution statement

**Shanlingzi Huang:** Conceptualization, Investigation, Writing – original draft. **Lu Gao:** Investigation, Methodology. **Yujun Chen:** Investigation, Methodology. **Zhaoming Fu:** Investigation. **Ziyou Wang:** Investigation. **Yifan Liu:** Methodology. **Zhicheng Zhou:** Investigation. **Ru Huang:** Funding acquisition, Methodology. **Wen Song:** Funding acquisition, Supervision, Writing – review & editing. **Feifan Zhou:** Funding acquisition, Supervision, Writing – review & editing.

## Declaration of competing interest

The authors declare that they have no known competing financial interests or personal relationships that could have appeared to influence the work reported in this paper.

## Data Availability

Data will be made available on request.
