# Supplementary material for: A versatile nanoplatform for enhancing the therapeutic efficacy against low-immunogenic TNBC by inducing immunogenic cell death and MHC-I upregulation
Source: Mater Today Bio. 2026 Feb 10;37:102927. doi: 10.1016/j.mtbio.2026.102927 (PMC12925286; doi:10.1016/j.mtbio.2026.102927)
Supplement: Multimedia component 1 [file mmc1.docx]

**A Versatile Nanoplatform for Enhancing the Therapeutic Efficacy against Low-Immunogenic TNBC by Inducing Immunogenic Cell Death and MHC-I Upregulation**

*Shanlingzi Huang ^a, b, 1^, Lu Gao ^a, b, 1^, Yujun Chen ^a, b, 1^, Zhaoming Fu ^a, b^, Ziyou Wang ^a, b^, Yifan Liu ^a, b^,* *Zhicheng Zhou ^a, b^, Ru Huang ^a, b^, Wen Song ^a, b, *^, Feifan Zhou ^a, b, *^*

^a^ State Key Laboratory of Digital Medical Engineering, School of Biomedical Engineering, Hainan University, Sanya 572025, China

^b^ Key Laboratory of Biomedical Engineering of Hainan Province, One Health Institute, Hainan University, Sanya 572025, China

**Corresponding Authors**

E-mail address: [songwen@hainanu.edu.cn](mailto:songwen@hainanu.edu.cn) (W. Song), [zhouff@hainanu.edu.cn](mailto:zhouff@hainanu.edu.cn) (F. Zhou)

^1^ These authors contribute equally to this work.

***1.Methods***

***1.1 Preparation of PCN-224, 3PNH and PH***

***PCN-224:*** 0.3 g of ZrOCl₂·8H₂O, 0.1 g of TCPP and 2.8 g of benzoic acid were dissolved in 100 mL of DMF. The mixture was continuously stirred under dark conditions at 90°C for 5 h. After the reaction, the product was collected by centrifugation at 12000 rpm for 20 min at room temperature, followed by washing with DMF three times. The resulting precipitate was resuspended in DMF and stored for further use.

***3PNH:*** 10 mg of PCN-224 and 50 mg of 3NPA were dispersed in 10 mL of deionized water and stirred under dark conditions for 12 h to achieve drug loading. The nanoparticles were collected by centrifugation and washed with deionized water three times. These nanoparticles were then mixed with a 1 mg/mL solution of hyaluronic acid and stirred for 3 h. Finally, the PCN@3NPA@HA (3PNH) nanoparticles were obtained after purification by centrifugation.

Drug loading efficiency(%)=$\frac{weight of the drug encapsulated in the nanoparticles}{total weight of the drug loaded nanoparticles}$×100%

Encapsulation efficiency(%)=$\frac{weight of the drug encapsulated in the nanoparticles}{initial weight of the drug}$×100%

***PH:*** The synthesis method of PCN@HA (PH) is identical to that of 3PNH. Specifically, a 1 mg/mL solution of HA was mixed with 10 mg of PCN-224 and stirred for 3 h. Subsequently, the mixture was centrifuged and washed with water to obtain PH.

***1.2 Cell Culture***

RPMI 1640 medium supplemented with 10% fetal bovine serum (FBS) was used to culture the 4T1 cell line under the conditions of 37℃, 5% CO_2_, and 95% air.

***1.3 Cytotoxicity Assay***

The dark cytotoxicity of PH or 3PNH for 4T1 cells was assessed using the CCK-8 assay after treatment with culture media containing various concentrations of PH or 3PNH for 24 hours. The photodynamic cytotoxicity of PH or 3PNH was evaluated by incubating 4T1 cells with culture media containing different concentrations of PH and 3PNH for 6 hours, followed by irradiation with a 660 nm laser (0.5 W/cm^2^) for 5 minutes. After further incubation for 18 hours, cell viability was measured using the CCK-8 assay.

***1.4 Characterization of ICD Induction in Tumor Cells by 3PNH***

4T1 cells were seeded at a density of 3×10^5^ per well in confocal dishes. After 24 hours of incubation, PBS, 3-NPA (6 μg/mL), PH (3 μg/mL), and 3PNH (3 μg/mL) were added respectively, followed by incubation for 6 hours. Subsequently, the cells were irradiated with a 660 nm laser (0.5 W/cm^2^) for 5 minutes and further cultured for 12 hours. The cells were then washed three times with PBS and subjected to the following treatments:

***CRT Staining:*** Cells were fixed with 4% PFA for 15 minutes and washed three times with PBS. The cells were incubated with the primary antibody (anti-CRT) at room temperature for 1 hour, washed with PBS, and then incubated with the secondary antibody at room temperature in the dark for 1 hour. Finally, the nuclei were stained with DAPI, and the expression of CRT protein in each group of cells was observed using a confocal laser microscope.

***HMGB1 Staining:*** Cells were fixed with 4% PFA for 15 minutes, permeabilized with 0.3% Triton X-100 for 30 minutes, and washed three times with PBS. The cells were incubated with the primary antibody (anti-HMGB1) at room temperature for 1 hour, washed with PBS, and then incubated with the secondary antibody at room temperature in the dark for 1 hour. Finally, the nuclei were stained with DAPI, and the expression of HMGB1 protein in each group of cells was observed using a confocal laser microscope.

***ATP Level Detection:*** Cells were collected and the intracellular ATP levels were measured according to the instructions of the ATP detection kit.

***1.5 Real-Time Quantitative PCR for Assessing MHC-I Expression in Tumor Cells***

4T1 cells were seeded at a density of 3×10^5^ cells per well in 6-well plates. After 24 hours of culture, the cells were co-cultured with PBS, 3NPA (30 μg/mL), PH (15 μg/mL) or 3PNH (15 μg/mL) for 24 hours. Total RNA was extracted from the cells and reverse-transcribed into cDNA. The expression levels of B2m, H-2k, H-2d, and the reference gene GAPDH were detected by reverse transcription according to the instructions of the TaKaRa kit. The relative expression levels of the genes were calculated using the 2^-ΔΔCt^ method(Analytik Jena).

The primers were designed as follows:

| B2m-F: | ACAGTTCCACCCGCCTCACATT |
| --- | --- |
| B2m-R: | TAGAAAGACCAGTCCTTGCTGAAG |
| H-2k-F: | GGCAATGAGCAGAGTTTCCGAG |
| H-2k-R: | CCACTTCACAGCCAGAGATCAC |
| H-2d-F: | TGAGGAACCTGCTCGGCTACTA |
| H-2d-R: | GGTCTTCGTTCAGGGCGATGTA |
| GAPDH-F: | AGGTCGGTGTGAACGGATTTG |
| GAPDH-R: | TGTAGACCATGTAGTTGAGGTCA |
|  |  |

1. ***Results***

|  | Drug Loading Efficiency (%) | Encapsulation Efficiency (%) |
| --- | --- | --- |
| 3PNH | 54.28 | 47.5 |

**Table. S1**. The drug loading and encapsulation efficiency of 3PNH.


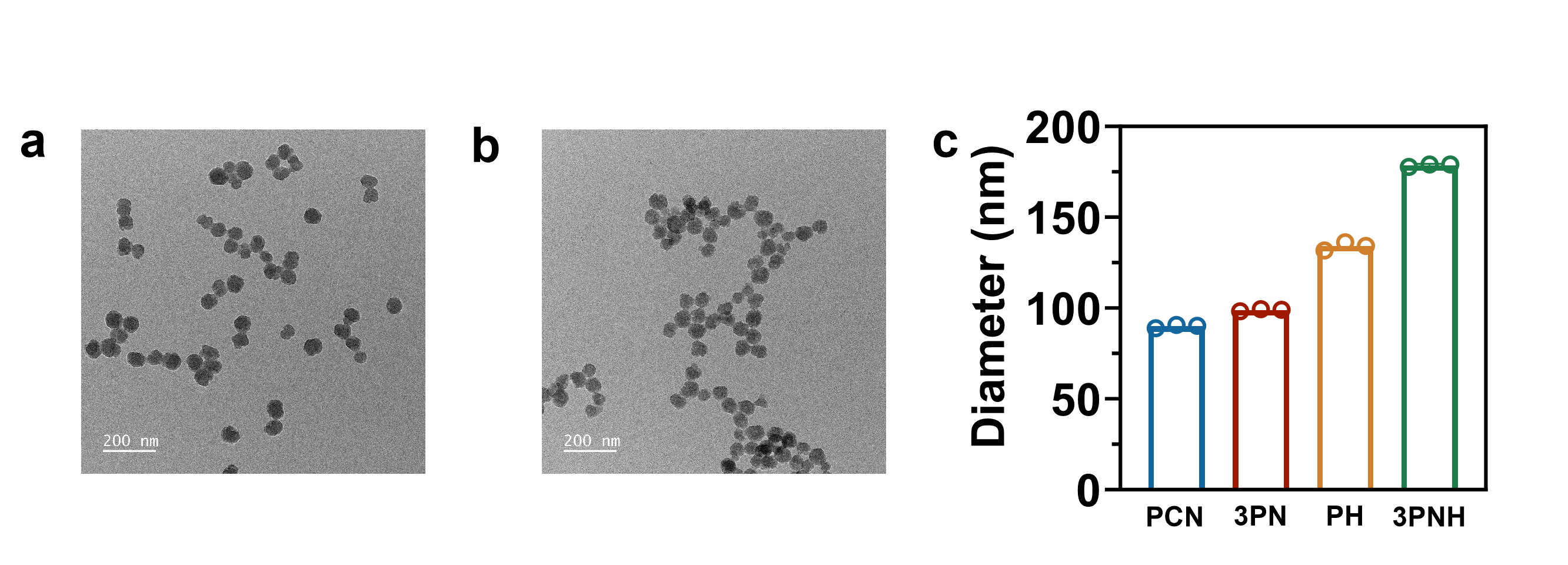


**Fig. S1**. The TEM image of **(a)** PCN-224 and **(b)** 3PNH **(c)** Hydrodynamic particle size of PCN, PH and 3PNH (n=3).


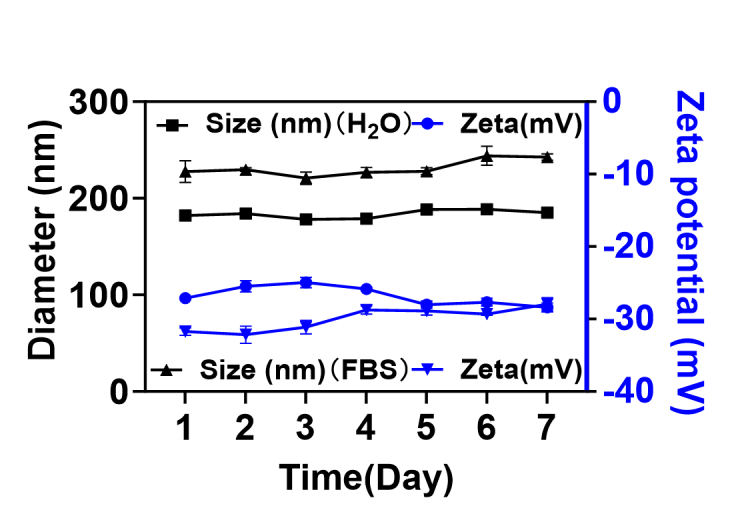


**Fig. S2**. Changes in hydrodynamic size and zeta potential of 3PNH dispersed in deionized water or 10% fetal bovine serum over 7 days (n=3).


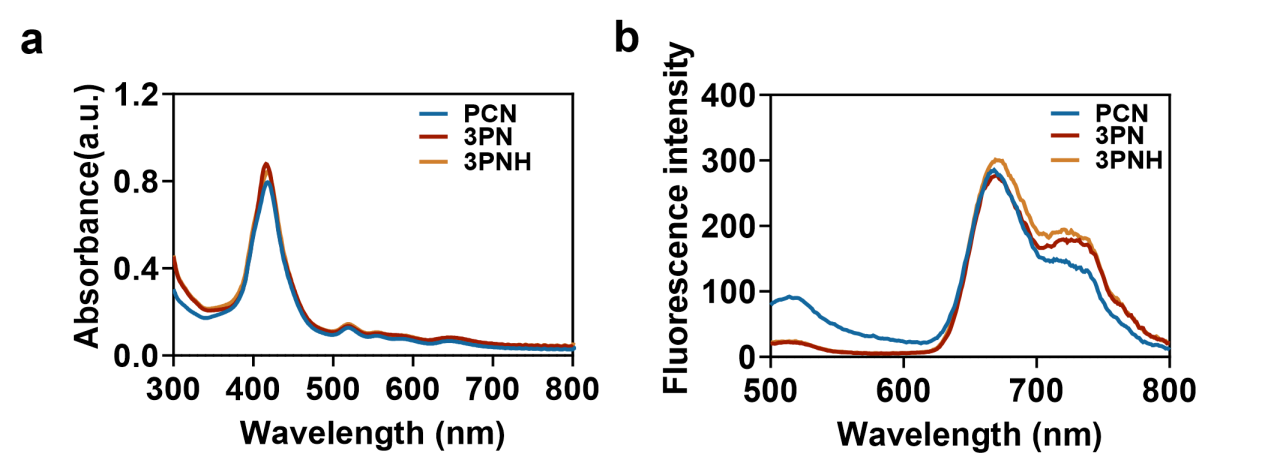


**Fig. S3**. (a) UV–vis absorption spectra and (b) fluorescence emission spectra of PCN, 3PN, and 3PNH.


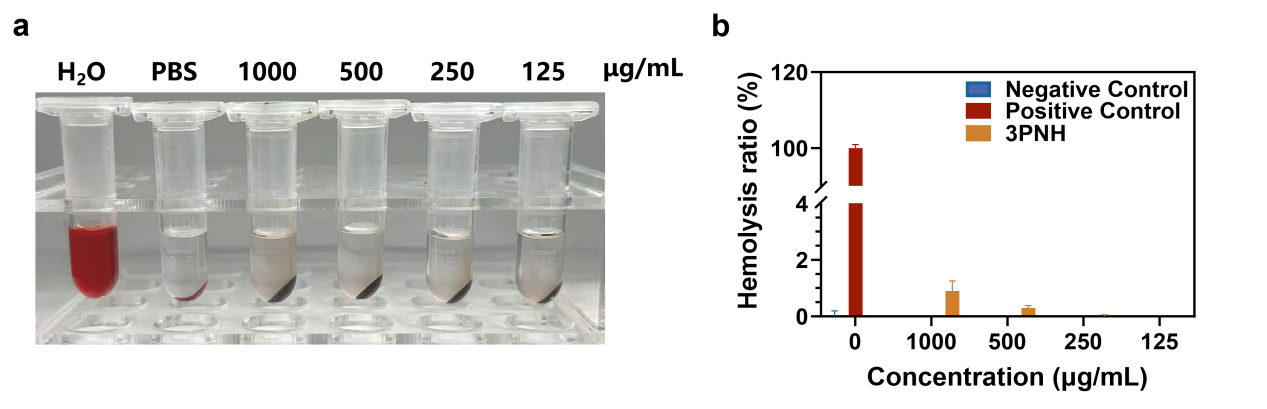


**Fig. S4**. (a) Images of hemolysis assays using PBS, H_2_O, and 3PNH at various concentrations; (b) Corresponding hemolysis rate statistics (n=3).


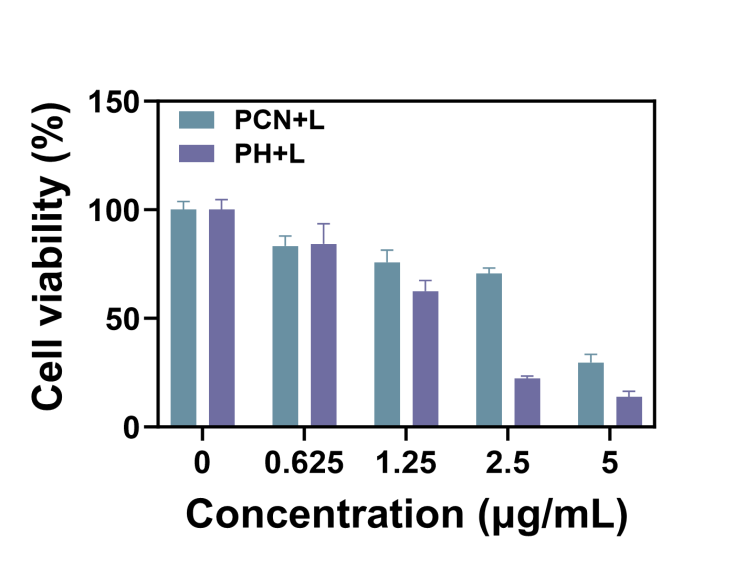


**Fig. S5.** Cell viability of 4T1 cells treated with PCN and PH under light irradiation (n=4).


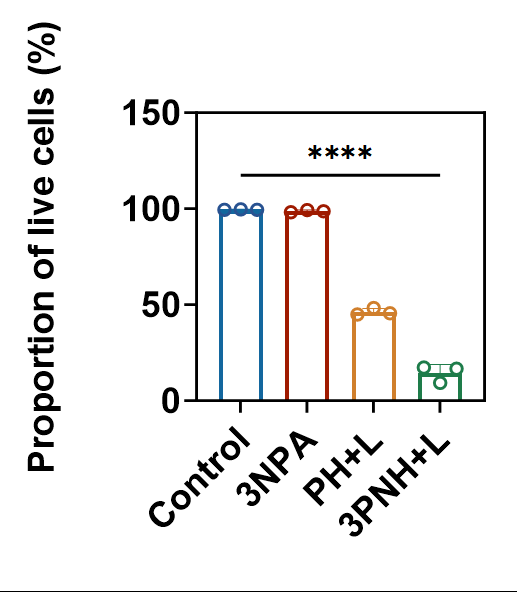


**Fig. S6**. Semi-quantitative analysis of cell viability in the Calcein-AM/PI assay (n=3).


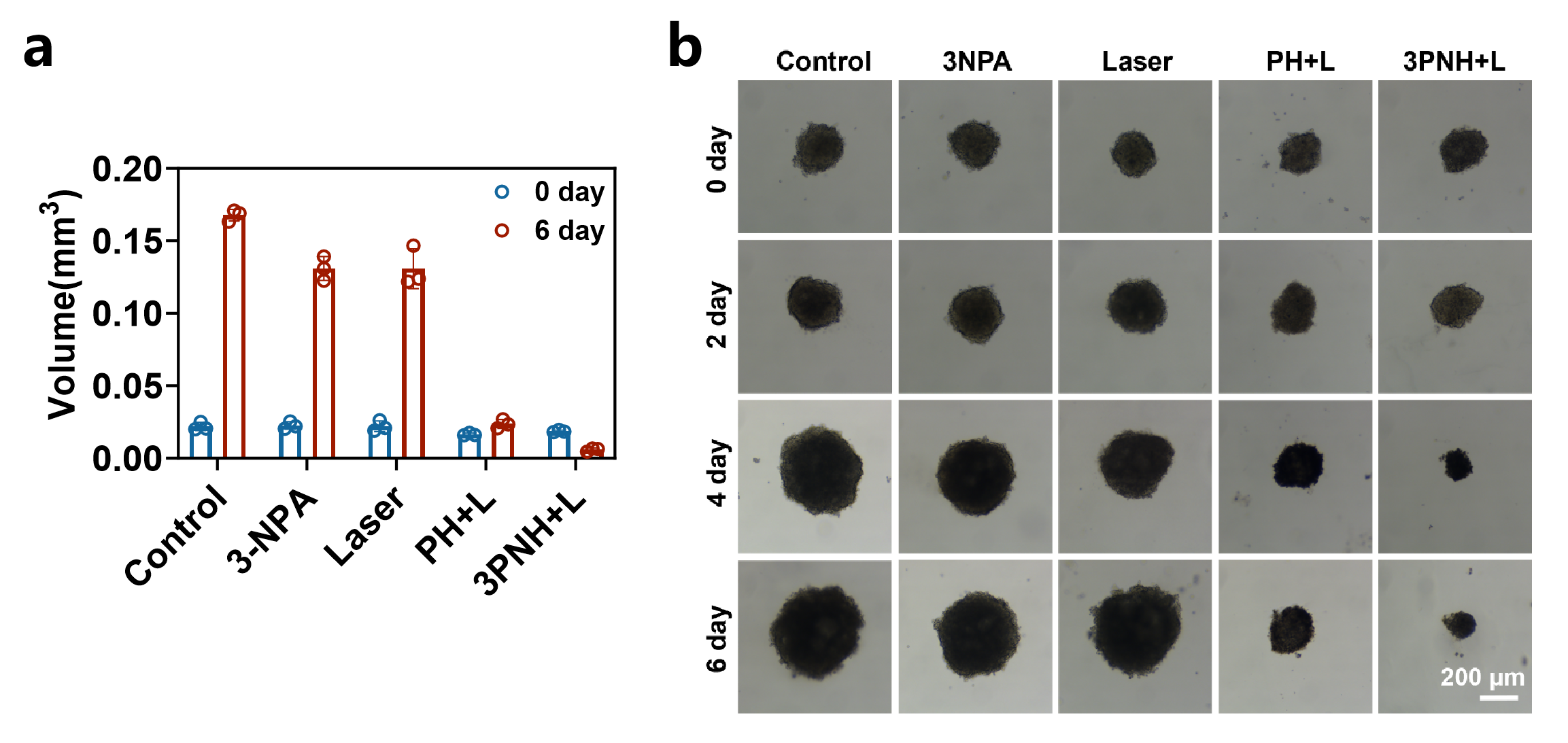


**Fig. S7**. (a) Volume changes of 3D tumor spheroids over 6 days under different treatment conditions (n=3). (b) Volume sizes of tumor spheroids on day 0 and day 6 under different treatment conditions.


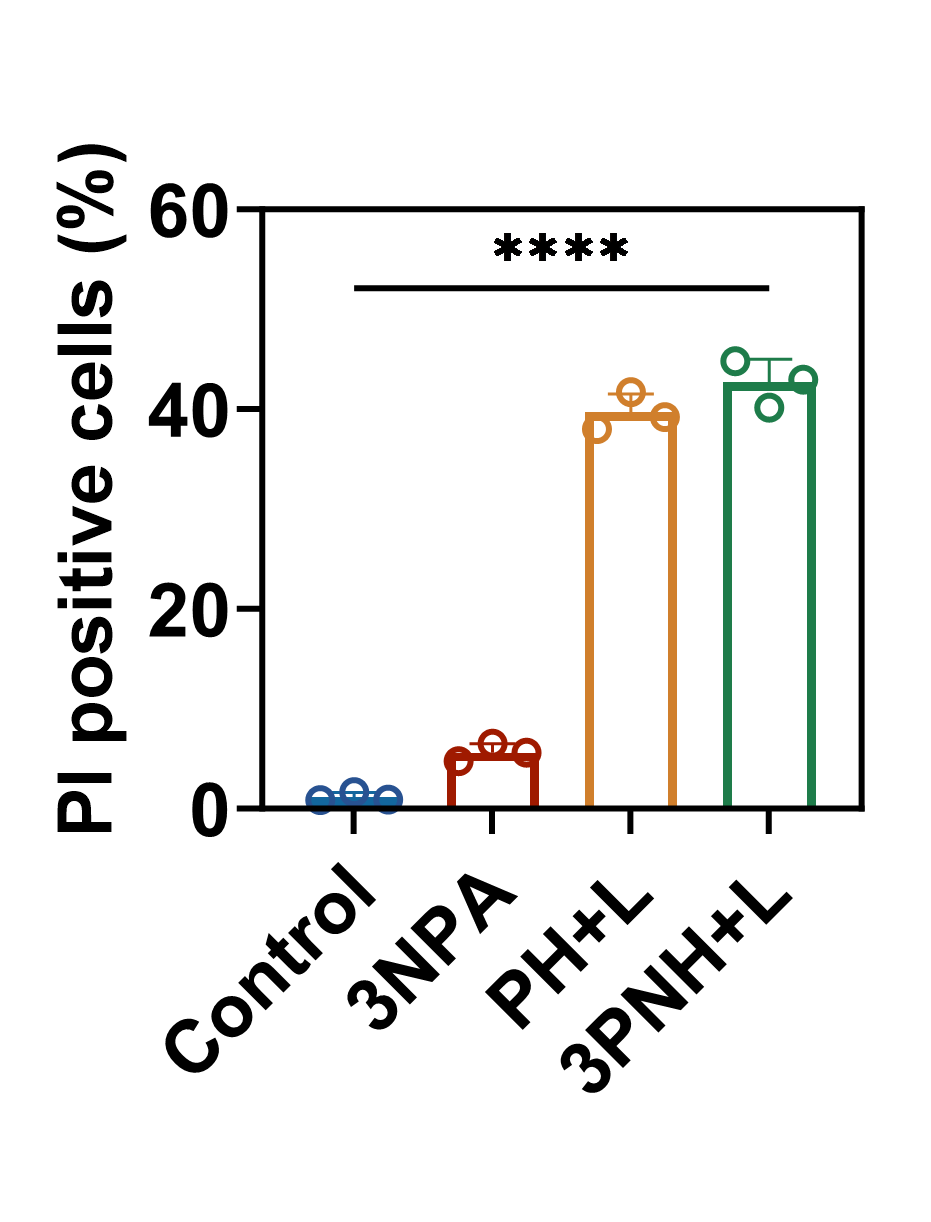


**Fig. S8**. Quantitative analysis of PI-positive tumor cells under different conditions in the apoptosis assay (n=3).

**Fig. S9**. Mean fluorescence intensity of TMRE in 4T1 cells under different treatment conditions(n=3).


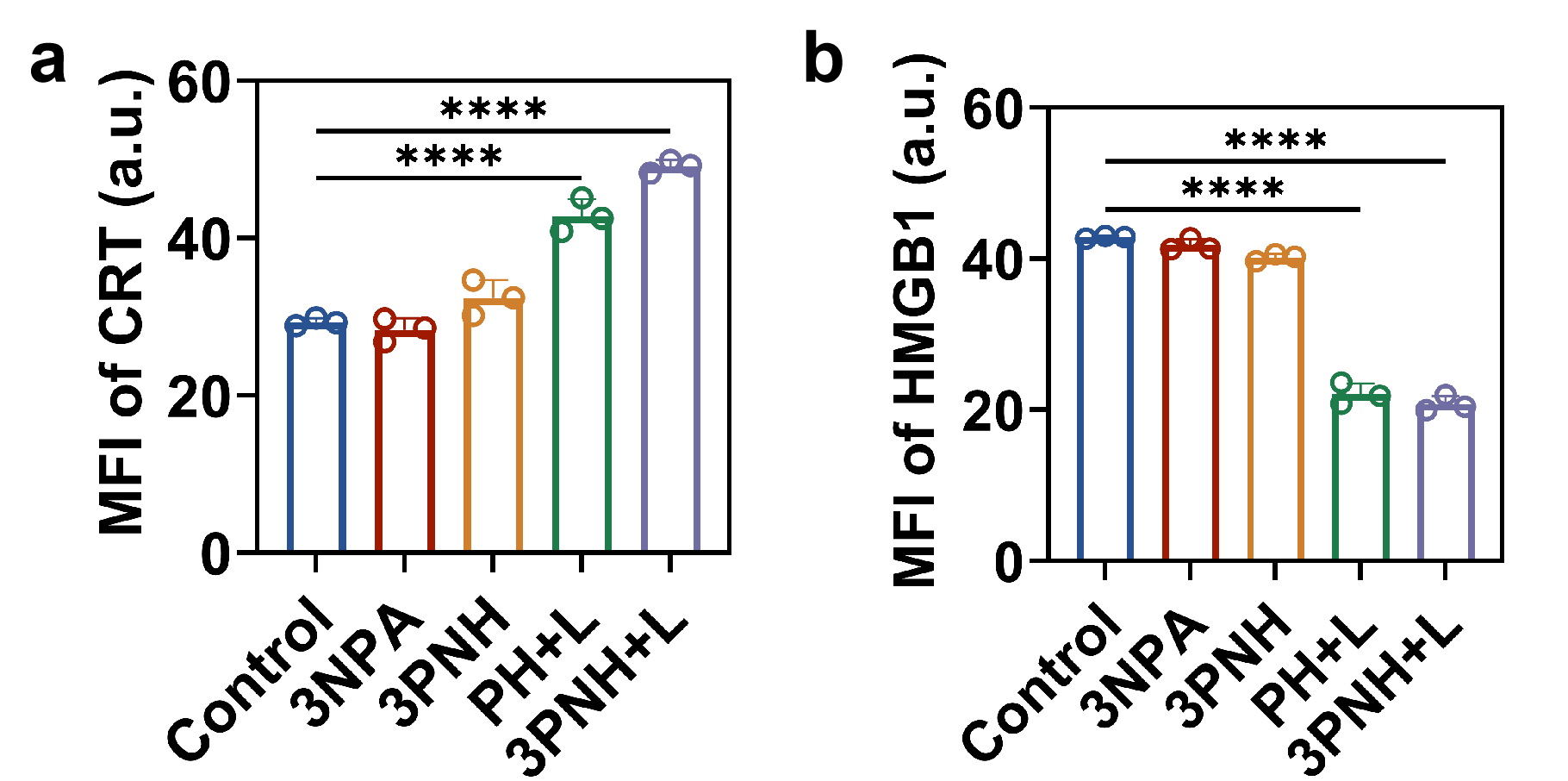


**Fig. S10**. Semi-quantitative analysis of the immunofluorescence results of (a) CRT and (b) HMGB-1 in 4T1 cells under different conditions (n=3).

**Fig. S11**. Quantitative assessment of MHC-I protein expression in 4T1 cells treated with different concentrations of 3NPA for 24 hours, as determined by flow cytometry (n=3).

**
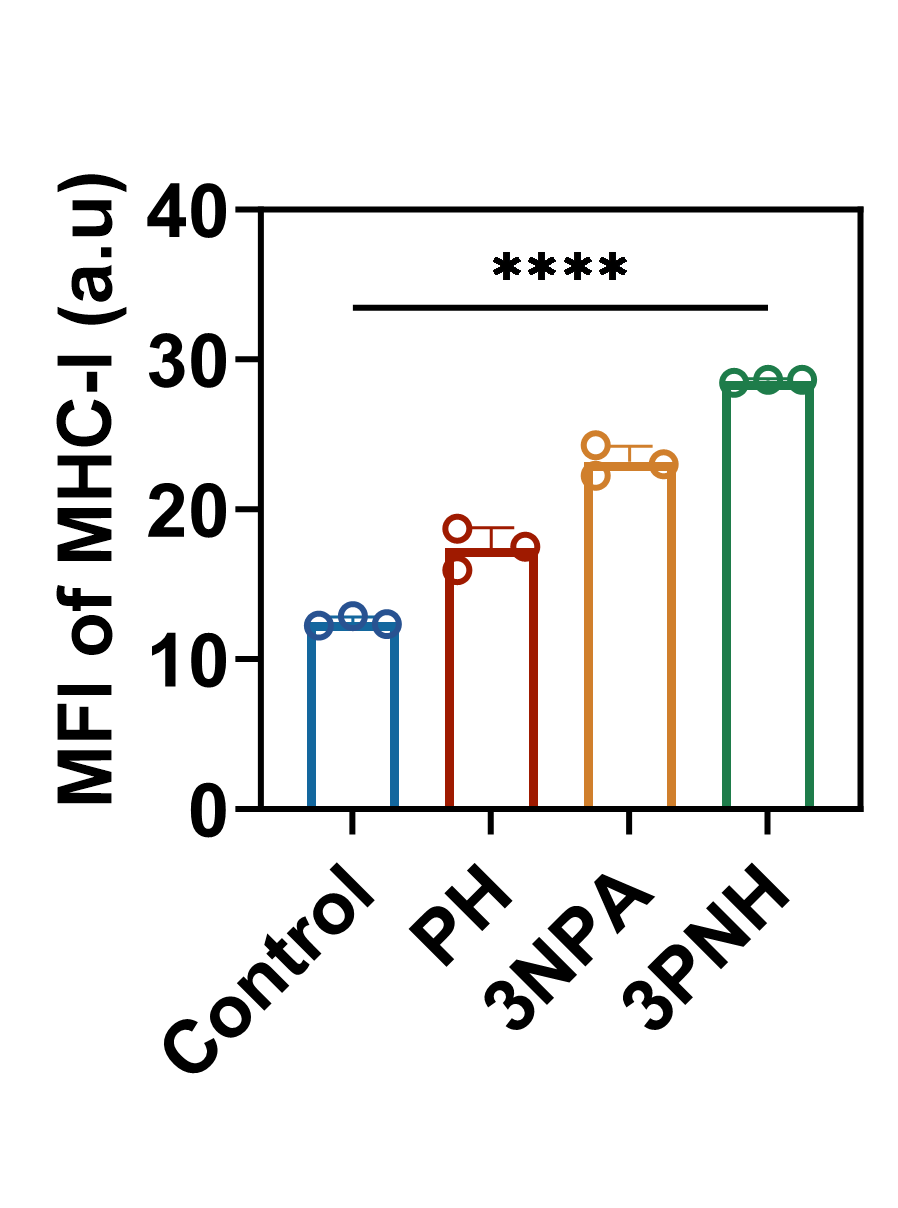
**

**Fig. S12**. Semi-quantitative analysis of MHC-I expression by immunofluorescence in 4T1 cells under different conditions (n=3).


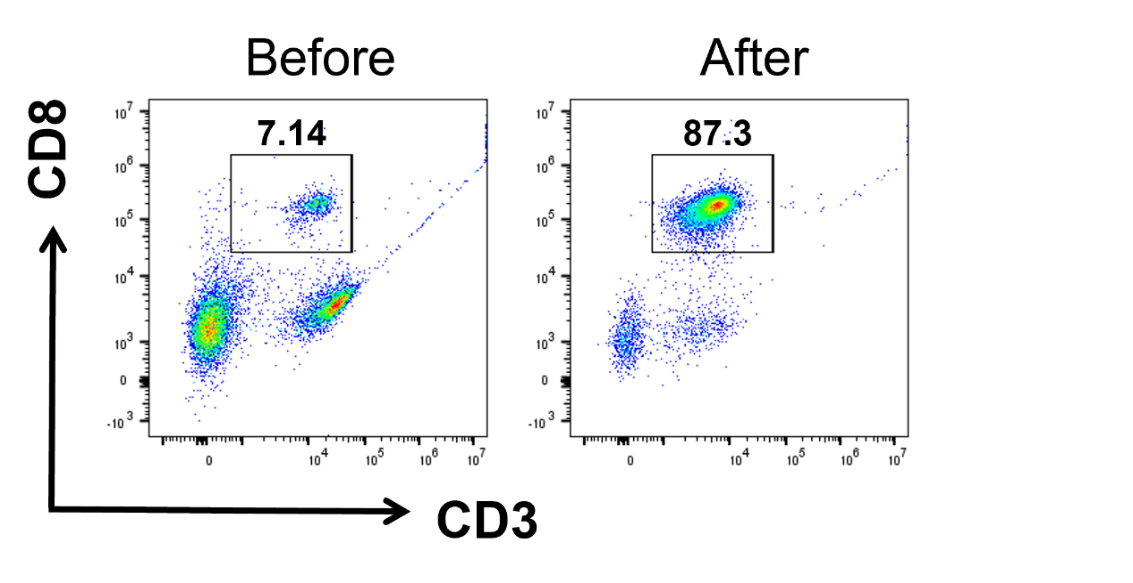


**Fig. S13**. Flow cytometric analysis of lymphocytes before and after using a mouse spleen lymphocyte separation kit.

**Fig. S14.** Flow cytometric quantitative analysis of CD3^+^CD8^+^CD107a^+^ cells after co-culture of 4T1 cells treated under different conditions with activated T lymphocytes (n=3).

**Fig. S15**. Changes in body weight of mice after different treatments over 12 days (n=5).


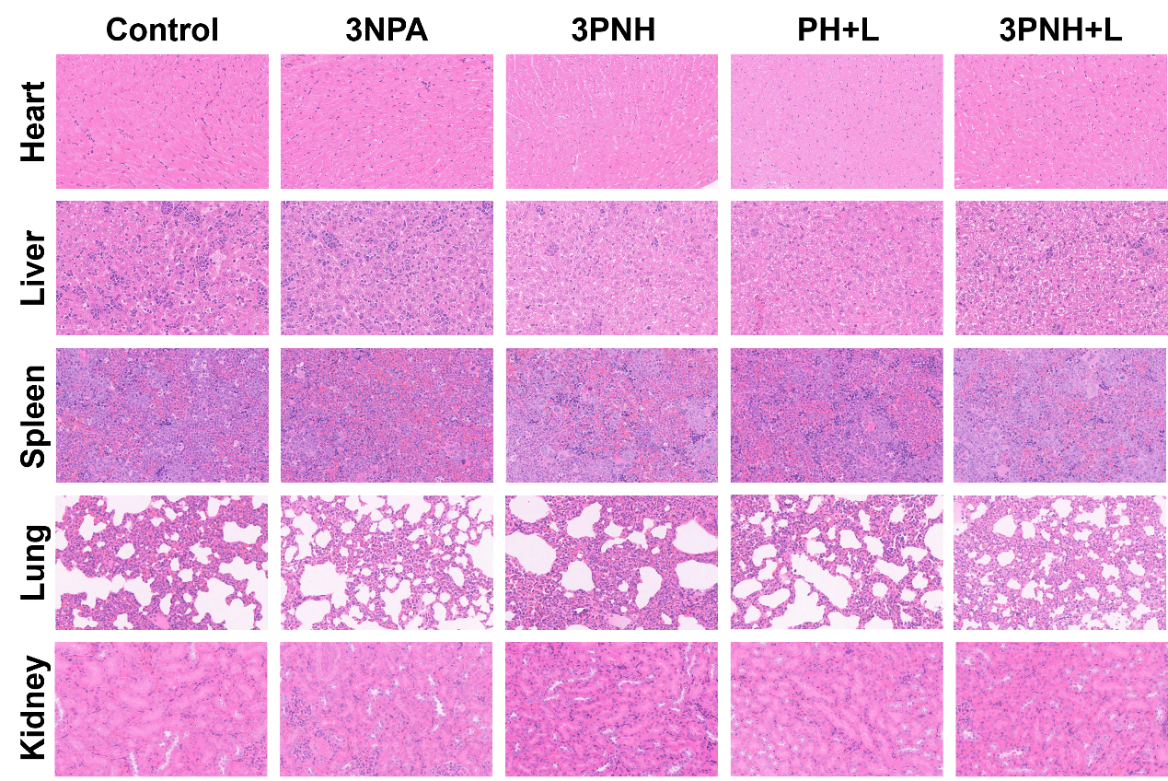


**Fig. S16.** H&E staining of major organs (heart, liver, spleen, lung, and kidney) from mice in each group after the completion of treatment.


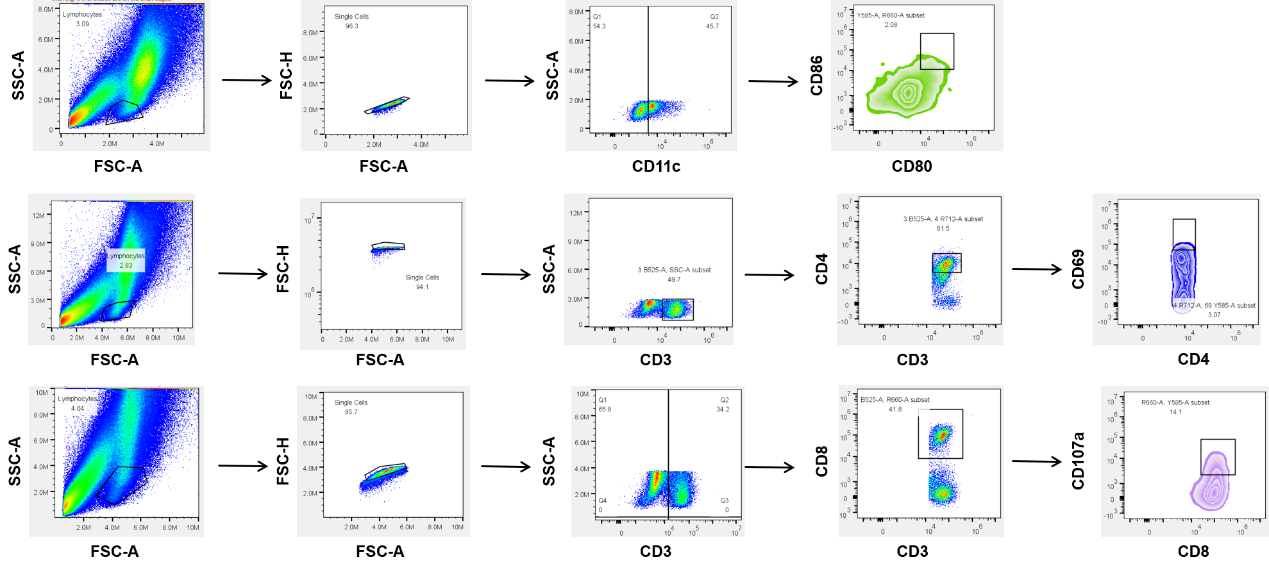


**Fig. S17** Gating strategy for flow cytometry presented in **Fig. 7**.
